# Shengxian decoction suppresses malignant progression of lung adenocarcinoma by enhancing CD8^+^ T cell function via the *FYN*-PI3K/AKT axis

**DOI:** 10.1186/s13020-026-01442-9

**Published:** 2026-07-06

**Authors:** Qiong Ma, Xiao Zeng, Chunxia Huang, Xingyue Liu, Jiawei He, Yuting Bai, Yuxuan Xiong, Shiyan Tan, Zongyi Mao, Xueke Li, Qian Wang, Xi Fu, Fengming You, Yifeng Ren, Chuan Zheng

**Affiliations:** 1 Hospital of Chengdu University of Traditional Chinese Medicine, No.39, Shierqiao Road, Jinniu District, Chengdu, 610075 China; 2 TCM Prevention and Treatment of Metabolic and Chronic Diseases Key Laboratory of Sichuan Province, Hospital of Chengdu University of Traditional Chinese Medicine, Chengdu, 610075 China; 3 Sichuan Provincal Engineering Technology Research Center of Natural Small Molecule Drug, Tianfu TCM Innovation Harbour, Chengdu University of Traditional Chinese Medicine, Chengdu, 611930 China

**Keywords:** Shengxian Decoction (SXD), Organoid, CRISPR/Cas9, Lung adenocarcinoma, FYN-PI3K/AKT axis

## Abstract

**Background:**

Shengxian Decoction (SXD), a traditional Chinese herbal formula, has demonstrated significant clinical efficacy against lung adenocarcinoma (LUAD). However, its exact mechanisms of action remain to be elucidated. This study aimed to establish an animal model capable of simulating the dynamic progression of LUAD to investigate the anti-tumor effects and immunomodulatory mechanisms of SXD.

**Methods:**

A TK (*Trp53*^*−/−*^; *Kras*^*G12D*^) LUAD mouse model was established by subcutaneously transplanting CRISPR/Cas9-edited mouse lung organoids into immunocompetent C57BL/6 mice. The model was characterized by histopathology, RT-qPCR, T7E1 assay, and transcriptomic analysis. Potential mechanisms of SXD were investigated through an integrated approach combining UHPLC-MS/MS, network pharmacology, molecular docking, and eQTL-based Mendelian randomization. Core targets and key signaling pathways were further evaluated by in vivo transcriptomic analysis, multiplex immunofluorescence, flow cytometry, an in vitro CD8^+^ T-cell coculture model, and surface plasmon resonance (SPR) analysis.

**Results:**

An immunocompetent TK-LUAD mouse model was successfully established, showing the dynamic pathological progression of LUAD and accompanying changes in immune infiltration. SXD treatment significantly delayed malignant progression in this model and was associated with modulation of the tumor immune microenvironment, as reflected by increased CD8^+^ T-cell infiltration and elevated levels of the cytotoxic effector molecules TNF-α, IFN-γ, and GZMB. Integrative analyses prioritized *FYN* as a candidate target potentially involved in SXD-mediated immunomodulation. SPR analysis showed concentration-dependent binding of representative SXD-derived compounds, including secoisolariciresinol, berberine, and acacetin, to *FYN*, providing additional support for direct target engagement. Further in vivo and in vitro experiments indicated that SXD enhanced the cytotoxic function of CD8^+^ T cells by activating the FYN-PI3K/AKT signaling pathway.

**Conclusion:**

This study establishes an immunocompetent animal model for investigating LUAD progression and associated changes in the tumor immune microenvironment. In addition, this study provides evidence suggesting that the antitumor activity of SXD may be associated with *FYN*-related activation of the PI3K/AKT signaling pathway and subsequent enhancement of CD8^+^ T cell function. These findings provide a theoretical and experimental basis for further investigation of SXD as a potential therapeutic strategy for LUAD.

**Graphical Abstract:**

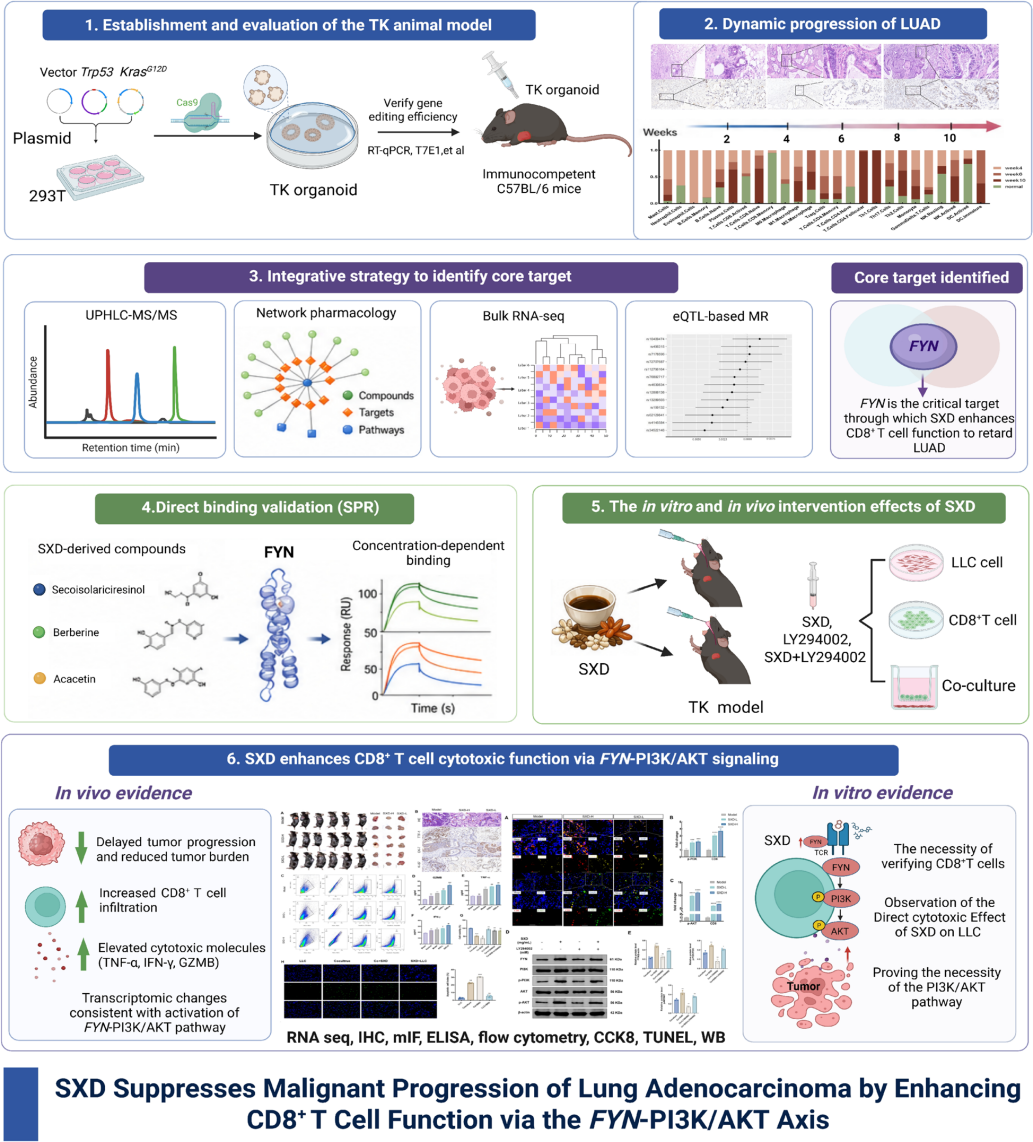

**Supplementary Information:**

The online version contains supplementary material available at 10.1186/s13020-026-01442-9.

## Introduction

As the predominant pathological subtype of lung cancer, lung adenocarcinoma (LUAD) has emerged as a major global public health challenge [1, 2]. Its pathogenesis involves a multi-stage evolutionary continuum, typically progressing histopathologically from atypical adenomatous hyperplasia (AAH) through minimally invasive adenocarcinoma (MIA) to invasive adenocarcinoma (IAC) [3, 4]. In the clinical setting, the rising detection of pulmonary nodules has provided mounting evidence that high-risk nodules act as precursor lesions for the early malignant transformation of LUAD [5, 6]. Consequently, unraveling the molecular mechanisms driving this malignant progression and developing effective early intervention strategies are urgent scientific priorities.

Mouse models serve as pivotal tools for elucidating the pathogenesis of LUAD and validating novel intervention strategies. While traditional chemical carcinogenesis models can induce pulmonary tumor formation, they are limited by high stochasticity and poor controllability, making it difficult to precisely replicate the linear progression of tumorigenesis [7]. Similarly, although genetically engineered mouse models (GEMMs) can simulate specific gene-driven tumorigenesis, they are often hampered by prolonged latency periods and low efficiency [8]. In recent years, organoid systems have emerged as a powerful tool for tumorigenesis research due to their superior controllability, genetic editability, and efficiency [9, 10]. *KRAS* and *TP53* are the most prevalent driver genes propelling the stepwise malignant progression of LUAD, and studies have confirmed that introducing these driver mutations into lung organoids can successfully model tumor transformation in vitro [11–13]. However, it remains unclear whether these transformed organoids, upon transplantation into immunocompetent mice, can faithfully recapitulate the complete evolutionary spectrum from precursor lesions to invasive carcinoma, while concurrently capturing the dynamic remodeling of the tumor immune microenvironment (TIME).

It is well established that malignant progression is accompanied by the dynamic remodeling of the TIME [14]. Particularly during the early malignant transformation of LUAD, the evolution of the TIME—characterized by alterations in immune cell infiltration and functional states—plays a pivotal role in tumor progression [15, 16]. Traditional Chinese Medicine (TCM), renowned for its multi-component, multi-target, and multi-pathway regulatory characteristics, has demonstrated unique potential in modulating the TIME and reversing immune suppression [17, 18]. Shengxian Decoction (SXD) was first recorded in the classic Chinese medical text, *Yi Xue Zhong Zhong Can Xi Lu*, by the renowned Qing Dynasty physician Zhang Xichun. The formula is composed of *Astragali Radix* (Huangqi, dried root of Astragalus membranaceus Fisch. ex Bunge), *Anemarrhenae Rhizoma* (Zhimu, dried rhizome of Anemarrhena asphodeloides Bunge), *Bupleuri Radix* (Chaihu, dried root of Bupleurum chinense DC.), *Platycodonis Radix* (Jiegeng, dried root of Platycodon grandiflorus A.DC.), and *Cimicifugae Rhizoma* (Shengma, dried rhizome of Cimicifuga heracleifolia Kom.) (pharmacological details refer to the Chinese Pharmacopoeia; botanical names verified via World Flora Online). Traditionally prescribed for “tonifying Qi and raising Yang,” SXD has been extensively used for centuries to treat pulmonary diseases, with modern pharmacology confirming its ability to enhance T cell- and B cell-mediated immune responses [19, 20]. Accumulating clinical and experimental evidence underscores its positive role in lung cancer prevention and treatment, including bolstering host immunity, improving patient quality of life, and inhibiting tumor cell proliferation [21–23]. These findings suggest that the formula may play a critical role in tumor immunomodulation. Consequently, there is an urgent need for systematic investigation to elucidate the potential mechanisms underlying SXD treatment in LUAD.

Accordingly, in the present study, we employed organoid culture combined with CRISPR/Cas9 gene-editing technology to establish an immunocompetent mouse model of LUAD driven by concomitant *Trp53* loss and *Kras* activation, aiming to faithfully recapitulate the linear progression of the disease. Building upon this platform, we further investigated the efficacy of SXD in suppressing LUAD progression and dissected the key molecular mechanisms by which it remodels the TIME and reinvigorates anti-tumor immune responses. Our findings are expected to provide a robust experimental foundation for the clinical application of TCM in the prevention and treatment of LUAD, while paving new avenues for the development of intervention strategies based on immunomodulation.

## Materials and methods

### Mice

ROSA26-CAG-Cas9-GFP transgenic mice were bred in our laboratory using stock originally acquired from The Jackson Laboratory (Cat# JAX:026179, RRID: IMSR_JAX:026179; male, 6–8 weeks old). For transplantation experiments, 6–8-week-old C57BL/6 mice were obtained from Gempharmatech Co., Ltd. The study protocol was reviewed and approved by the Animal Ethics Committee of the Hospital of Chengdu University of Traditional Chinese Medicine (Approval No. 2024DL-016).

### Cell culture

The human embryonic kidney 293T (HEK293T) cells required for the experiments were purchased from the Cell Resource Center of the Chinese Academy of Sciences.

Lewis lung cancer (LLC, Anwei-sci Cell Center, M0084) cells were obtained from the Cell Bank of the Shanghai Institute of Cell Biology (China). CD8⁺ T cells were isolated from fresh mouse spleens using magnetic bead sorting (130-116-478, Miltenyi Biotec, China). The isolated cells were seeded into plates pre-coated with anti-CD3 (10 μg/mL) and stimulated with anti-CD28 (4 μg/mL) and IL-2 (10 ng/mL). After 24 h of activation, half of the culture medium was replaced, and the cells were continuously expanded for use in subsequent experiments. LLC cells were cultured in DMEM (Abiowell, AW-M003, China) supplemented with 10% FBS (EvaCell, E01010, China) and 1% penicillin–streptomycin (P/S) (Abiowell, AWH0529a, China). CD8^+^ T cells were maintained in RPMI 1640 (Abiowell, AW-M002, China) medium containing 10% FBS and 1% P/S. All cells were incubated in a humidified atmosphere at 37 ℃ with 5% CO₂. For drug treatments, the LY294002 inhibitor (MCE, HY-10108, USA) and SXD lyophilized powder were prepared at working concentrations of 20 μM and 1 mg/mL, respectively. For the inhibitor-rescue group, CD8^+^ T cells were pre-treated with 20 μM LY294002 for 4 h. Following a wash step to remove the inhibitor, the cells were treated with 1 mg/mL SXD for 24 h, and subsequently co-cultured with LLC cells for an additional 24 h. For the SXD treatment group, CD8^+^ T cells were treated with 1 mg/mL SXD for 24 h prior to being co-cultured with LLC cells for 24 h.

### Mouse organoid culture

Mouse lung tissues were harvested, minced into approximately 3 mm^3^ fragments, and digested in DMEM/F12 containing 1.0 mg/mL collagenase I (Gibco, 17100-017) and 0.5 mg/mL collagenase IV (Gibco, 17104-019) at 37 °C for 45 min. The resulting suspension was filtered through a 100-μm strainer, centrifuged, and washed with DMEM/F12 (Gibco, C11330500BT). The cell pellet was then resuspended in Matrigel, seeded into culture plates, and allowed to polymerize at 37 °C. Upon solidification, 150 μL of organoid expansion medium (ExM) was added to each well. The ExM and growth factors were kindly provided by Prof. Chen (Department of Thoracic Oncology, State Key Laboratory of Biotherapy and Cancer Center, West China Hospital, Sichuan University, Chengdu, Sichuan 610041, China) [24]. Organoids were passaged every 5–7 days using TrypLE(Gibco, 12605-028).

### CRISPR/Cas9-mediated organoid transfection and functional verification

The CRISPR/Cas9-mediated *Trp53* knockout vector was constructed based on the V2T-mCherry system. In this construct, a U6 promoter drives the expression of an sgRNA targeting the conserved domain of *Trp53* exon 5, while an EFS promoter regulates the mCherry reporter gene; an optimized sgRNA scaffold was incorporated to ensure high editing efficiency. For the *Kras* activation vector, the pMSCV retroviral system was employed to engineer an expression vector for the oncogenic *Kras*^*G12D*^ (c.35G > A) mutant. This construct utilizes an IRES element to enable co-expression with the Luc2 reporter gene, alongside a WPRE element to enhance post-transcriptional regulation. Vectors were assembled via Gibson Assembly and transformed into E. coli DH5α competent cells. Positive clones were screened based on antibiotic resistance, verification of reporter gene fluorescence, and confirmation by Sanger sequencing, thereby completing the plasmid construction.

### Luciferase reporter assay

293T cells at 48 h post-packaging or organoids at 48 h post-infection were selected. When the cell density and condition were appropriate, the viral supernatant or culture medium was discarded, and the cells were washed once with 500 ul of PBS. Subsequently, 60 ul (for 6-well plates) or 10 ul (for 48-well plates) of Firefly luciferase substrate solution was added. The luminescence signals were detected using a multimode microplate reader to obtain the values, followed by data analysis.

### Organoid transplantation

Organoids were dissociated into single cells using TrypLE express 800 μl at 37 °C for 10–15 min, with intermittent pipetting. Cells were counted and resuspended in a 1:1 mixture of PBS and Matrigel. Subsequently, 2× 10^5^ cells (in 200 μL total volume) were injected subcutaneously into the left posterior axillary line of 6–8-week-old C57BL/6 mice using a 1 mL insulin syringe.

### Bioluminescent imaging

Mice were given 150 mg/kg D-luciferin potassium salt (Biovision, 7903–10PK) intraperitoneally and imaged on the IVIS spectrum i*n vivo* imaging system (PerkinElmer).

### In vivo treatment

Mice were assigned to five experimental groups: Blank normal (Blank, n = 6), Control (Control, n = 10), Model (Model, n = 10), Shengxian Decoction high-dose (SXD-H, n = 10), and Shengxian Decoction low-dose (SXD-L, n = 10). Treatments were administered via oral gavage at a volume of 0.1 mL/10g body weight. The Blank, Control, and Model groups received an equivalent volume of physiological saline. The day of transplantation was designated as Day 0; drug administration was initiated on Day 1 and terminated after 10 weeks.

### H&E, immunohistochemistry and immunofluorescence staining

For histopathological staging, H&E-stained sections obtained at weeks 4, 6, and 10 after transplantation were independently evaluated by two pathologists blinded to the time points. Lesion classification was performed using criteria adapted from WHO/IASLC histologic principles for lung adenocarcinoma progression. The classification was based on the overall architectural pattern, degree of epithelial stratification, cytologic atypia, necrosis, and immunophenotypic findings, including TTF-1 (HUABIO, HA720067, 1:1000), CK7 (HUABIO, ET1609-62, 1:1000), and Ki-67 (Abcam, ab15580, 1:1000) expression. Interobserver agreement was assessed using Cohen’s kappa coefficient, and discrepant cases were resolved by joint review to reach a consensus diagnosis. Immunofluorescent staining was performed using the antibodies CD68 (ImmunoWay, YM8067, 1:1000), p-PI3K (ImmunoWay, YM8723, 1:1000), and p-AKT (ImmunoWay, YM8531, 1:1000). Images were captured using a Nikon ECLIPSE C1 camera.

### RNA extraction and RT–qPCR

Total RNA was isolated using TRIzol (Applied Biosystems, 15596026) and reverse-transcribed into cDNA using M-MLV reverse transcriptase (Invitrogen, 28025013), following the manufacturers’ protocols. RT–qPCR assays were conducted on a QuantStudio 3 system (Applied Biosystems) utilizing Powerup SYBR green master mix (Applied Biosystems, A25741). Relative gene expression was calculated via the 2^−ΔΔCt^ method and normalized to actb or hprt. All reactions were performed in triplicate.

### Western blot (WB) analysis

Total proteins from CD8^+^ T cells were extracted using RIPA lysis buffer (AWB0136, Abiowell, China), and protein concentrations were determined using a BCA protein assay kit (AWB0104, Abiowell, China). The protein samples were loaded onto sodium dodecyl sulfate-polyacrylamidegel electrophoresis gels (SDS-PAGE) gels and then transferred to a 0.45 μm PVDF membrane. PVDF membrane was immersed in TBST containing 5% skim milk and blocked at RT on a shaker for 1.5 h, then incubated with specific primary antibodies on a shaker overnight at 4 ℃. The PVDF membrane was immersed in the secondary antibody [Goat anti-Rabbit IgG (H + L) Secondary Antibody, HRP, Abiowell, China, 1:5000] incubation solution for 1.5 h after being rinsed with TBST three times, 10 min for each time. After incubation with the ECL reagent (a mixture of solution A and B in equal proportion), the membranes were visualized by imaging system (iBright 750, Invitrogen, USA).

### Flow cytometry

Tumor tissues were harvested and dissociated into single-cell suspensions. Cells were incubated with anti-CD4 antibody (Abcam, ab237722, 1:200), Anti-CD8 antibody (Abcam, ab217344, 1:200) at 4 °C for 30 min in the dark. Data acquisition was performed on a BD LSRFortessa flow cytometer (BD Biosciences), and data were analyzed using FlowJo software.

### ELISA

Peripheral blood was collected via retro-orbital puncture and centrifuged to isolate serum. The concentrations of TNF-α (Wuhan Fine Biotech Co., Ltd., EM0183), IFN-γ (Wuhan Fine Biotech Co., Ltd., EM0093), and GZMB (Shanghai Jianglai Biotechnology Co., Ltd, JL11913) were measured using mouse ELISA kits according to the manufacturer’s protocols. Absorbance at 450 nm was measured using a microplate reader (PerkinElmer, USA). Sample concentrations were calculated by interpolation from standard curves. All samples were analyzed in duplicate, and the experiment was repeated at least three times.

### Cell viability assay

Log-phase cells were digested, quantified, and seeded into 24-well plates at a density of 1 × 10^4^ cells/well. Upon adherence, the cells were subjected to the specified treatments for the appropriate time periods. The Cell Counting Kit-8 (CCK-8; DOJINDO, NU679, Japan) solution was prepared by diluting the reagent in complete medium at a 1:10 ratio (10 μL CCK-8 per 100 μL medium). The original culture medium was discarded, and 300 μL of the CCK-8 working solution was added to each well. Following an additional 4-h incubation at 37 °C with 5% CO₂, the supernatant was collected and transferred to a 96-well plate with three technical replicates per group. Finally, the absorbance was recorded at 450 nm utilizing a microplate reader.

### RNA-seq analyses

RNA-seq libraries were constructed using an Illumina stranded mRNA sample preparation kit (NEB, E7770) according to the manufacturer’s protocol and were sequenced on an Illumina NovaSeq 6000 sequencing machine with 150-base pair (bp) paired-end reads. The RNA-seq reads were aligned to the mouse reference genome (GRCm38) by STAR_2.6.0a. Transcript abundance was normalized and measured by transcripts per kilobase million. DESeq2 (v.1.26.0, RRID:SCR_000154) was used to identify differentially expressed genes. Genes with an absolute log2(fold change) greater than 0.5 and a *P* value of < 0.05 were counted as differentially expressed genes. Pheatmap (v.1.0.12, RRID:SCR_016418) was used to display heat maps of the expression levels of differentially expressed genes, which were normalized by z score.

### External data analysis

Screening and visualization of differentially expressed genes (DEGs) from the Gene Expression Omnibus (GEO) Database. Gene expression datasets related to early-stage lung adenocarcinoma were retrieved from the GEO database. Using the search terms "early stage lung adenocarcinoma" and "Stage I & Stage IIA," datasets GSE118370 and GSE140797 were identified. Samples were categorized into a Control group (healthy individuals) and a disease group (LUAD patients). DEGs were screened using the limma package in R software. The selection criteria were set as |log_2_FC|> 0.585 and adjusted *P* < 0.05. Finally, the identified DEGs were visualized using volcano plots and heatmaps.

### eQTL analysis of exposure data

The TwoSampleMR R package was utilized to process eQTL data and identify single nucleotide polymorphisms (SNPs) significantly associated with target gene expression as instrumental variables (IVs). The selection of IVs adhered to the following criteria: (1) a genome-wide significance threshold (*P* < 5 × 10⁻⁸); (2) linkage disequilibrium (LD) clumping (window size = 10 Mb, *r*^*2*^ < 0.001) to ensure independence; and (3) validation of instrument strength (F-statistic > 10).

### Mendelian randomization (MR) analysis

A two-sample MR framework was established using standardized eQTL data to investigate causal associations [25], ensuring sample independence to minimize overlap bias. Analyses were performed using the TwoSampleMR package, primarily utilizing the Inverse Variance Weighted (IVW) method. Statistical significance was defined as a Benjamini–Hochberg adjusted *P* < 0.05. Genes exhibiting inconsistent effect directions across five MR methods (IVW, Weighted Median, MR-Egger, Simple Mode, and Weighted Mode) were excluded. Robustness was validated via sensitivity analyses: (1) Cochran’s Q test for heterogeneity; (2) MR-Egger intercept test to assess horizontal pleiotropy (where *P* > 0.05 indicates no significant pleiotropy); (3) leave-one-out analysis; and (4) MR-PRESSO for outlier correction.

### Identification of active compounds and target prediction

The active compounds identified via UHPLC-MS/MS were screened based on ADME (absorption, distribution, metabolism, and excretion) parameters in the TCMSP database, with criteria set as oral bioavailability (OB) ≥ 30% and drug-likeness (DL) ≥ 0.18. Subsequently, potential drug targets were retrieved from the SwissTargetPrediction, ETCM, and TCMSP databases by combining the UHPLC-MS/MS results with database screening and literature verification.

### Molecular docking

AutoDock Vina (http://vina.scripps.edu/) was employed to prepare the ligands and proteins for molecular docking. The crystal structures of the target proteins were retrieved from the Protein Data Bank (PDB, https://www.rcsb.org/). These structures underwent preprocessing, which included the removal of water molecules, modification of amino acid residues, energy optimization, and adjustment of force field parameters. The chemical structures of the ligands were downloaded from PubChem (https://pubchem.ncbi.nlm.nih.gov/) and optimized to obtain their low-energy conformations. Subsequently, molecular docking between the target structures and the active ingredients was performed using the built-in Vina engine of PyRx software (https://pyrx.sourceforge.io/). The binding affinity (kcal/mol) was used to evaluate the binding capability; a lower binding energy indicates a more stable interaction between the ligand and the receptor. Finally, the docking results were visualized and analyzed using PyMOL (https://pymol.org/2/).

### Surface plasmon resonance (SPR) analysis

SPR binding assays were performed using a Biacore 8K system (Cytiva, USA). Purified FYN protein was immobilized onto a CM5 sensor chip via amine coupling. The protein coupling buffer consisted of 1.0 × PBS-P + (pH 7.4), and the interaction buffer was 1.0 × PBS-P + (pH 7.4) containing 5% (v/v) DMSO. After immobilization, test compounds were serially diluted in running buffer and injected over the chip at flow rates of 30 μL/min in order of increasing concentration. The association and dissociation phases were both set to 60 s. After each analytical cycle, the chip was regenerated with 10 mM glycine–HCl solution (pH 2.0). Data were globally fitted to a 1:1 Langmuir binding model using Biacore Insight Evaluation software to calculate the association rate constant (*k*_*a*_), dissociation rate constant (*k*_*d*_), and equilibrium dissociation constant (*K*_*D*_).

### Target prioritization and multiple-testing control

Candidate targets were prioritized through integration of multiple predefined lines of evidence: (1) differentially expressed genes associated with LUAD identified from public datasets; (2) targets potentially related to SXD screened via network pharmacology analysis; (3) genes supported by MR based on eQTLs and validated through external public databases; and (4) biological relevance to tumor immunity, particularly associations with CD8^+^ T cell infiltration and function. Molecular docking results between active SXD compounds and candidate targets served as supplementary mechanistic evidence. Among the overlapping core targets, prioritization was performed based on effect size, robustness, cross-cohort consistency, and correlation with immune cells.

### Statistical analysis

Where applicable, multiple comparisons were corrected using the Benjamini–Hochberg method to control the false discovery rate (FDR). Adjusted P values were used for significance assessment, with FDR < 0.05 considered statistically significant. Statistical analysis and graphing were performed using SPSS 28.0 and GraphPad Prism 8.0 software. Data are presented as mean ± standard deviation (mean ± SD). Differences between groups were analyzed using Student’s t-test or one-way analysis of variance (ANOVA). Statistical significance is indicated as follows: *P < 0.05, **P < 0.01, ***P < 0.001.

## Results

### Construction and validation of a *Trp53/Kras*-driven lung premalignant organoid

#### Establishment and characterization of TK organoids

**Fig. 1:**
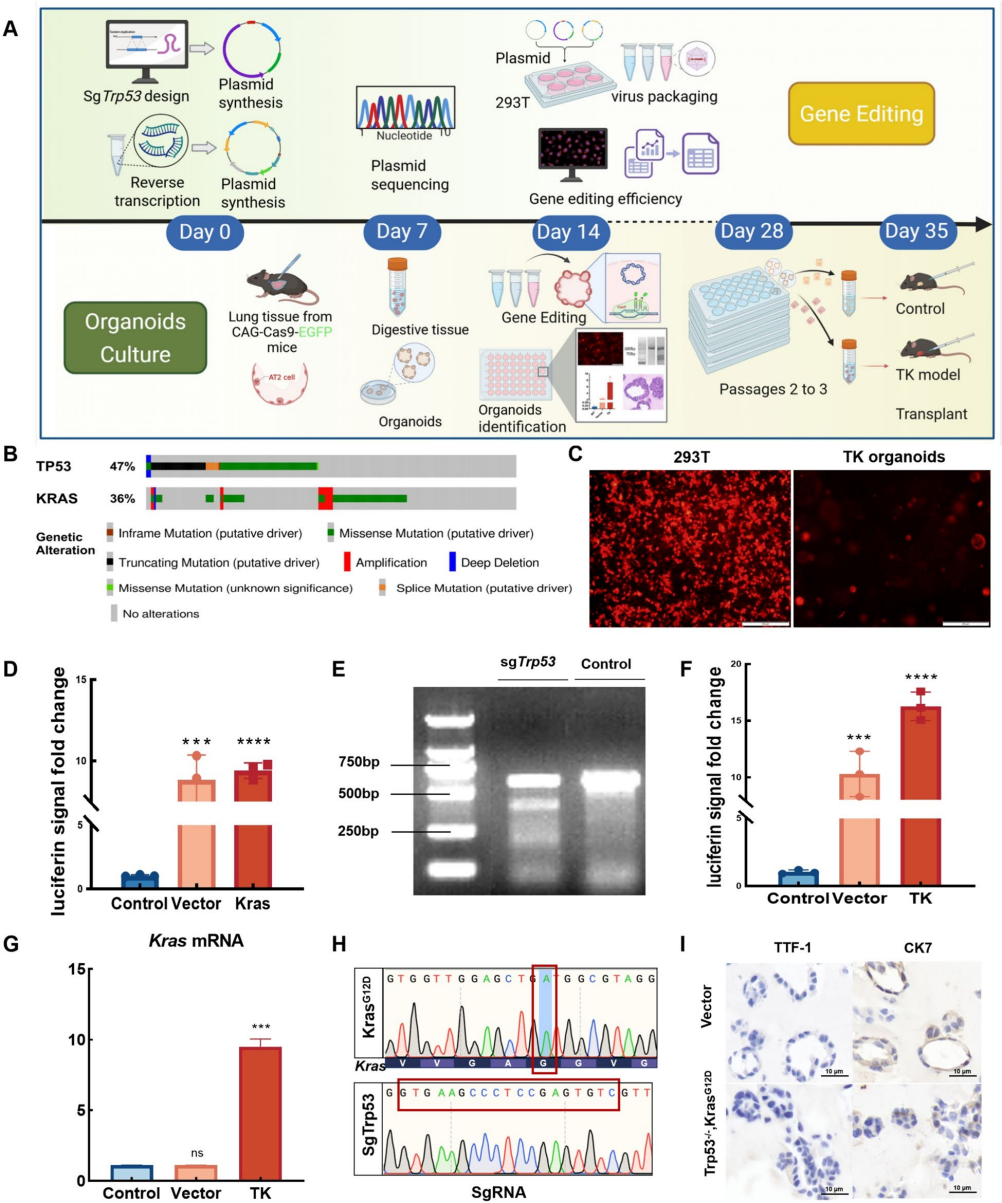
Establishment of TK organoids and validation of gene editing efficiency. **A** Schematic of the TK organoid design and in vivo transplantation workflow. **B** OncoPrint showing the variation frequencies of *TP53*, *KRAS* in 1590 LUAD samples from the cBioPortal dataset. **C** Fluorescence images of 293T cells transfected to produce viral particles and mouse lung organoids transduced with the resulting viral vectors. Scale bar, 200 μm. **D** Luminescence signal intensity of control, Vector and *Kras* 293T. **E** T7 endonuclease 1 (T7E1) assays on *Trp53* using infected organoids from **C**. **F** Luminescence signal intensity of control, Vector and *Kras* organoids. **G** *Kras* mRNA expression levels in control, vector, and TK organoids. Data are shown as mean ± SD. ***P < 0.001, ****P < 0.0001, calculated by *p*-value was calculated by unpaired t-test. **H** Gene sequencing peak map of *Kras* and *Trp53* in plasmid constructs. The box indicates the 12th amino acid and its codon in *Kras* gene (top), as well as the sg*Trp53* guide RNA sequence (bottom). **I** IHC staining of TTF-1 and CK7 in vector and TK organoids. Scale bar, 10 µm

To establish a genetic model capable of recapitulating clinical LUAD initiation, we analyzed genomic data from 1,590 primary LUAD samples retrieved from the cBioPortal for Cancer Genomics database (http://cbioportal.org). Our analysis identified *TP53* (47%) and *KRAS* (36%) as the two most frequently mutated driver genes, with the *G12D* substitution representing a predominant subtype within the *KRAS* mutant population. Notably, a significant co-occurrence of mutations in these two genes was observed in LUAD (Fig. 1B). Building upon these findings, we engineered a TK (Trp53^−/−^; *Kras*^*G12D*^) organoid model using the CRISPR/Cas9 system. Lung organoids derived from alveolar type 2 (AT2) cells of CAG-Cas9-EGFP mice were transduced with lentiviral vectors encoding *sgTrp53* and retroviral vectors expressing *Kras*^*G12D*^ (Fig. 1A).

Subsequently, we validated the efficiency of gene editing by comparing the TK model with wild-type (Control) and empty vector (Vector) groups. Initially, the stable expression of mCherry confirmed the successful transduction of the sgTrp53-carrying lentiviral vector into the organoids (Fig. 1C, D), while the T7E1 assay further verified the successful disruption of the *Trp53* locus (Fig. 1E). Luciferase reporter assays revealed that bioluminescence signals in both the Vector and TK groups were higher than in the Control group; notably, the TK group exhibited significantly higher intensity than the Vector group (Fig. 1F). RT-qPCR analysis demonstrated a significant upregulation of *Kras* mRNA levels in the TK group compared to controls (Fig. 1G). Crucially, sequencing analysis visualized via the Integrative Genomics Viewer (IGV) precisely confirmed the specific G > A mutation within the *Kras* gene (Figs. 1H and S1). Furthermore, prior to in vivo transplantation, we assessed the expression of standard LUAD markers. Immunohistochemical analysis revealed that TK organoids were negative for both TTF-1 and CK7. Collectively, these data demonstrate the successful editing of both *Trp53* and *Kras* genes in the lung organoids.

#### TK organoids exhibit early malignant transformation phenotypes in vitro

**Fig. 2:**
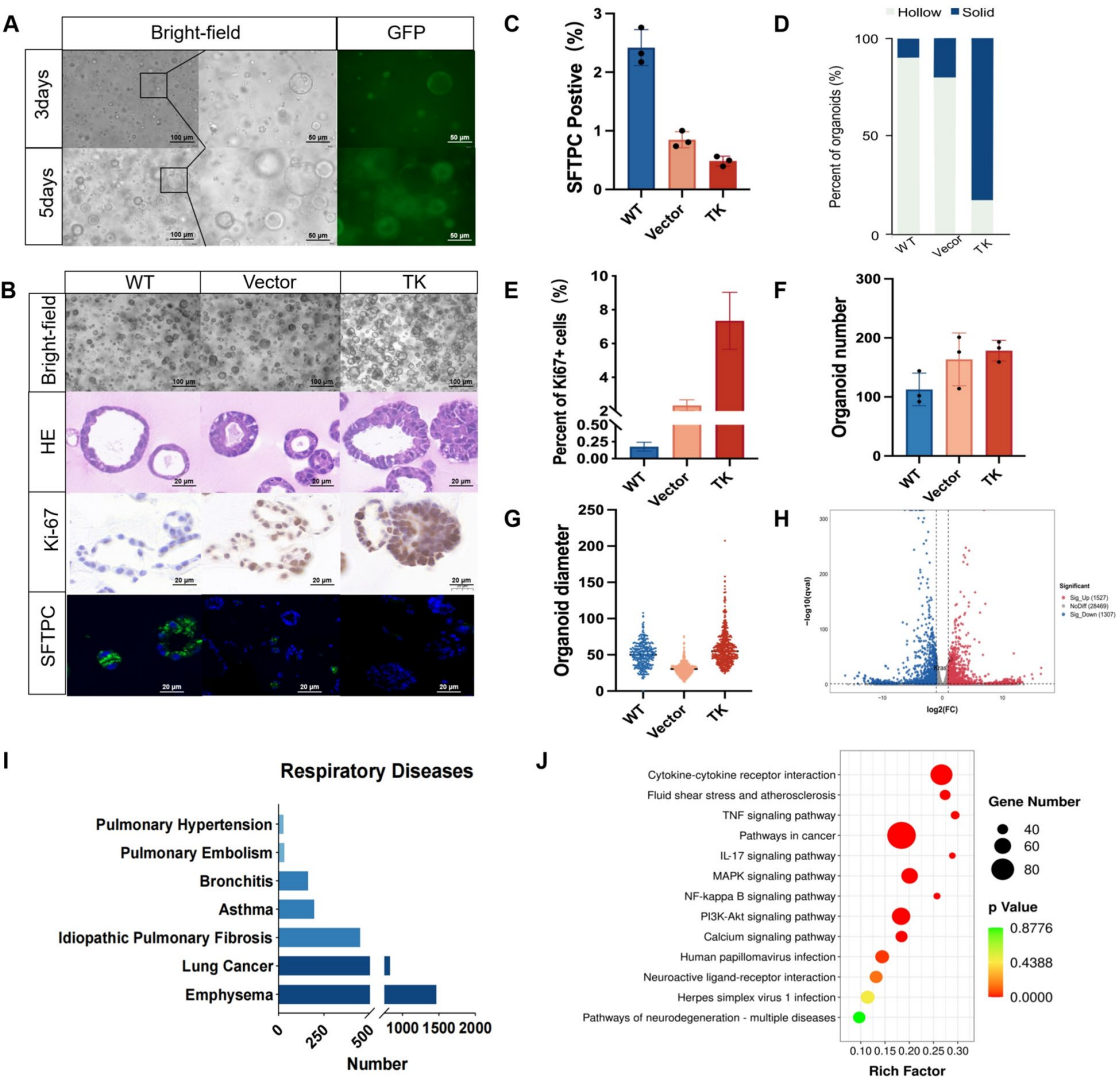
Generation and validation of *Trp53*/*Kras*-driven pre-adenocarcinoma lung organoids. **A** Representative bright-field and green fluorescent images of the normal lung organoids on day 3 and day 5. Scale bar, 50 μm. **B** Representative bright-field images and H&E, IHC and immunofluorescence staining of Ki-67, SFTPC in WT, vector, TK (*Trp53*^−/−^; *Kras*^*G12D*^) mice lung organoids. Scale bar, 200 μm, 50 μm, 50 μm, 50 μm. **C** The percentages of SFTPC + cells in WT vector, TK lung organoids. Data presented as the means ± SD; measured with Image J software, p value was calculated by unpaired t test. **D** The percentages of hollow and solid organoids in WT, vector, TK lung organoids. **E** The percentages of Ki-67^+^ cells in WT, vector, TK lung organoids. Data presented as the means ± SD; measured with Image J software, p value was calculated by unpaired t test. **F** The number of WT, vector, TK lung organoids. Data presented as the means ± SD; p value was calculated by unpaired t test. **G** The diameters of WT, vector, TK lung organoids. Data presented as the means ± SD; measured with Image J software, p value was calculated by unpaired t test. **H** Volcano plot displaying differentially expressed genes between the vector and TK organoids. **I** Gene Ontology analysis showing respiratory disease–related enriched genes in TK organoids. **J** Kyoto Encyclopedia of Genes and Genomes (KEGG) analysis showing pathways enriched in TK organoids

Gene editing induced significant morphological alterations in the organoids. By day 5 post-transduction (Fig. 2A), Control organoids exhibited typical cystic structures, and immunofluorescence confirmed their predominant composition of AT2 cells (Fig. 2B and C). In contrast, the Vector control group displayed mild epithelial stratification, whereas TK organoids formed disorganized, solid cell clusters (Fig. 2B and D). Ki-67 staining and quantification revealed that the proliferation index in TK organoids was significantly higher than that in both WT and Vector groups (Fig. 2B and E). Additionally, TK organoids exhibited a marked increase in diameter (Fig. 2F and G), indicating that concurrent *Trp53* deletion and *Kras*^*G12D*^ mutation collectively drive enhanced proliferation.

#### Transcriptomic analysis reveals molecular signatures of malignant transformation in TK organoids

To confirm the molecular differences underlying the malignant transformation trend between Vector and TK organoids, we performed transcriptome sequencing on both groups (n = 3). Compared to the Vector group, 2,834 DEGs were identified in TK organoids, including 1,527 upregulated and 1,307 downregulated genes (Fig. 2H). GO enrichment analysis indicated that these DEGs were significantly enriched in biological processes related to respiratory diseases, with 'lung cancer'-related terms showing strong enrichment (Fig. 2I). To further elucidate changes in key signaling pathways, KEGG pathway analysis revealed the significant enrichment of multiple known oncogenic and inflammatory pathways, including the TNF, IL-17, MAPK, NF-κB, and PI3K/AKT signaling pathways (Fig. 2J). These transcriptomic data suggest that concurrent *Trp53* and *Kras* mutations drive early malignant transformation by activating key proliferative and immuno-inflammatory pathways through extensive gene expression reprogramming.

### TK organoids recapitulate the stepwise progression of LUAD in immunocompetent mice

#### Establishment and evaluation of the TK animal model

**Fig. 3:**
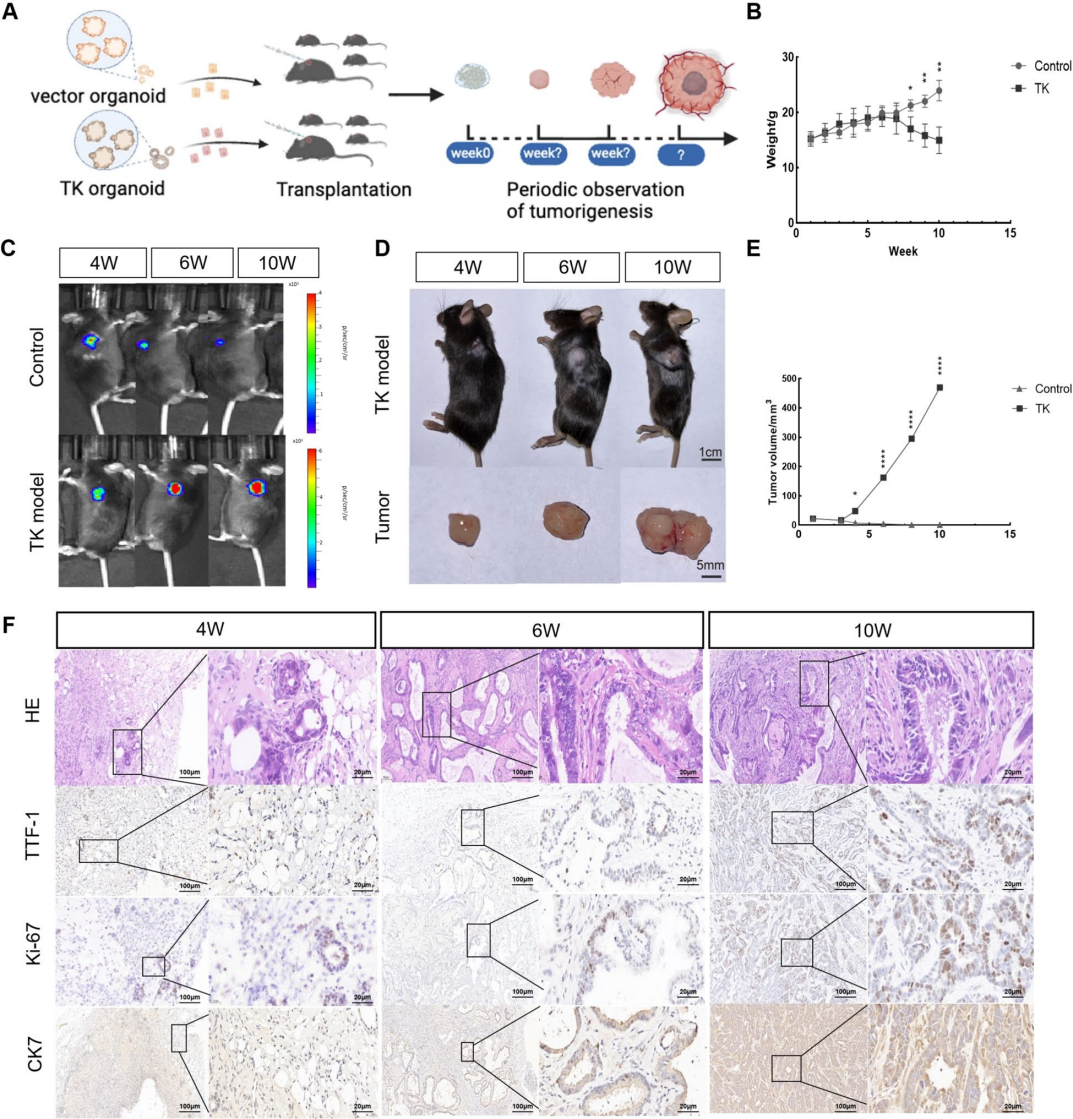
Stepwise histologic evolution of LUAD-like lesions initiated by genome-edited lung organoids in immunocompetent mice. **A** Schematic illustration of the transplantation procedure of TK organoids into immunocompetent mice. **B** Body weight curves of the control group (n = 3) and TK groups (n = 3). Data presented as the means ± SD; *P < 0.05, **P < 0.01, *p* value was calculated by unpaired t test. **C** Representative bioluminescence images of TK mice at weeks 4, 6, and 10 after transplantation. **D** Representative bright-field images of the mouse bodies and the subcutaneous tumors. **E** The growth curve of subcutaneous TK tumors (n = 3). Data presented as the means ± SD; ***P < 0.001, ****P < 0.0001, *p* value was calculated by unpaired t test. **F** Representative pictures showing H&E and IHC stainingsof TTF-1, CK7, and Ki-67 in tumor sections of the TK mice at week4, week6, week10. Scale bar, 100 μm, 20 μm

To evaluate the tumorigenicity of *Trp53*-deficient and *Kras*-mutant organoids in immunocompetent mice and their capacity to recapitulate disease progression, TK organoids were subcutaneously transplanted into C57BL/6 mice (Fig. 3A and B). Notably, organoids in the Vector group were rapidly cleared by the host immune system post-transplantation, failing to form tumors. In sharp contrast, the TK organoid group exhibited a 100% tumor incidence (Table S1).

Furthermore, the transplanted TK tumors displayed distinct phase-specific growth kinetics. During the first 4 weeks after transplantation, the tumors underwent an apparent adaptation phase characterized by slight regression, possibly reflecting adaptation to the host microenvironment and/or a transient local inflammatory response. This was followed by a slow-growth phase from weeks 4 to 6, and then by a rapid progression phase after week 6, accompanied by a marked increase in intratumoral fluorescence intensity (Fig. 3C–E). Based on these growth dynamics, weeks 4, 6, and 10 were selected as representative time points for pathological evaluation.

Histopathological evaluation revealed progressive acquisition of malignant histologic features over time. Given that this model is based on subcutaneous transplantation rather than orthotopic lung tumorigenesis, lesion classification was performed using histomorphologic criteria adapted from WHO/IASLC principles for lung adenocarcinoma progression[4], together with immunophenotypic support from TTF-1, CK7, and Ki-67 staining [26]. Specifically, the week 4 lesions were classified as AAH-like precursor-like lesions, showing focal epithelial proliferation with mild cytologic atypia, relatively simple architecture, and a low Ki-67 index; these cells were CK7 and TTF-1 positive. The week 6 lesions were classified as early adenocarcinoma-like lesions, characterized by increased epithelial stratification, mild-to-moderate nuclear atypia and an elevated Ki-67 index. By week 10, the lesions exhibited features of high-grade invasive adenocarcinoma-like tumors, including marked cellular atypia, loss of polarity, focal necrosis, diffuse positivity for TTF-1 and CK7, and a substantially increased Ki-67 index, indicating highly proliferative activity (Fig. 3F). All sections were independently reviewed by two pathologists in a blinded manner, and interobserver agreement was assessed using Cohen’s kappa (κ = 0.85).

#### Dynamic molecular landscape and immune microenvironment features of TK-LUAD progression

**Fig. 4:**
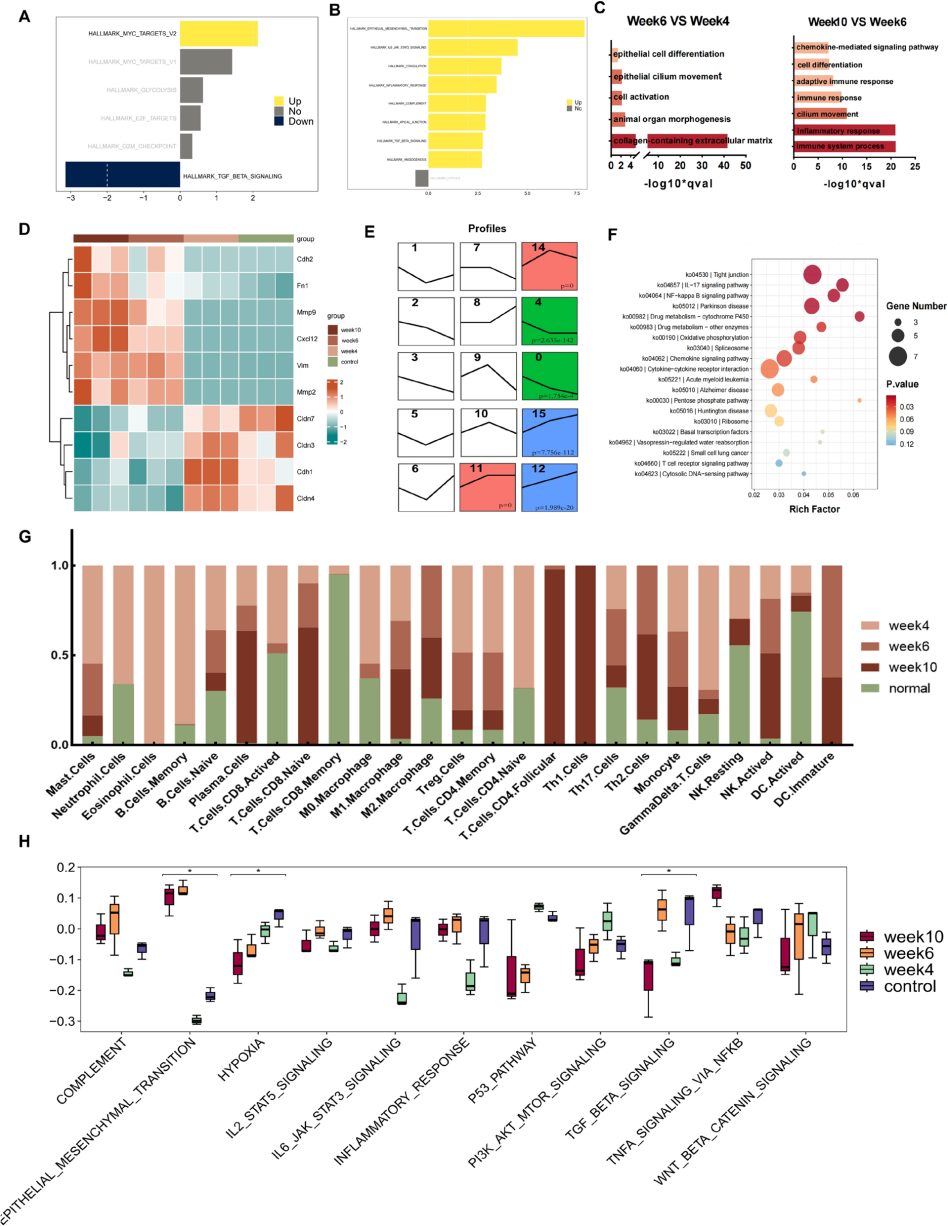
Transcriptomic analysis of progressive features in the TK model.** A** GSVA of Hallmark gene sets showing differential enrichment in TK model between week 6 and week 4. **B** GSVA of Hallmark gene sets showing differential enrichment in TK model between week 10 and week 6. **C** Gene Ontology enrichment analysis reveals temporal functional changes in TK mice, comparing week 6 vs week 4 and week 10 vs week 6. **D** Heatmap showing significantly altered epithelial–mesenchymal transition (EMT)–related genes in TK model tumors at week 4, week 6, and week 10, as well as in normal controls. **E**, **F** Short Time-series Expression Miner showing clustering of DEGs across week 4, week 6, and week 10 in the TK model tumors (**E**) and KEGG enrichment of Cluster 12 (**F**). **G** boxplot showing enrichment changes of CIBERSORT immune-infiltration–related pathways in tumors of TK model at week 4, week 6, and week 10, as well as in normal controls. *P < 0.05. **H** barplot showing relative proportions of immune cell types in TK model tumors at week 4, week 6, and week 10, as well as in normal controls

To delineate the dynamic molecular events driving LUAD initiation and progression, we performed transcriptomic analysis on tumor tissues at different stages. The early transition phase (Week 6 vs. Week 4) was characterized by extracellular matrix remodeling and epithelial differentiation, indicating active proliferation accompanied by stromal alterations. Conversely, the malignant progression phase (Week 10 vs. Week 6) exhibited a distinct shift towards inflammatory responses and oncogenic signaling, including the IL6/JAK/STAT3, TGF-β, and EMT pathways (Figs. 4A–C, S2). Consistent with the alterations in signaling pathways, a characteristic evolution of the EMT molecular phenotype was observed: As the disease progressed, the expression of epithelial markers (e.g., *Cdh1*, *Cldn3/4/7*) was gradually downregulated, while mesenchymal and migration markers (e.g., *Cdh2*, *Fn1*, *Vim*) and invasion-associated molecules (*Mmp2*, *Mmp9*) showed a time-dependent upregulation (Fig. 4D). These findings demonstrate that the TK model effectively recapitulates the molecular transition from early proliferation to late-stage invasion typical of human LUAD.

Furthermore, dynamic clustering analysis of DEGs was performed using the STEM algorithm. This analysis identified a total of 15 gene clusters exhibiting distinct temporal expression profiles. Notably, Cluster 12 demonstrated a statistically significant trend of continuous upregulation throughout disease progression (*P* < 0.05; Fig. 4E). Subsequent KEGG pathway enrichment analysis revealed that genes within Cluster 12 were significantly enriched in the NF-κB signaling pathway, the IL-17 signaling pathway, and pathways related to innate immunity (Fig. 4F).

Concurrently, tumor progression was accompanied by the dynamic remodeling of the TIME. Analysis of immune cell infiltration across the three time points identified 25 distinct immune cell types and functions. Regarding cellular composition, Week 4 was characterized by B cell infiltration and an upregulated Type I IFN response, indicative of early immune recognition. By Week 6, the environment shifted towards immune activation, evidenced by increased infiltration of activated DCs, CD8^+^ T cells, Th1 cells, and NK cells, alongside elevated CCR levels. However, by Week 10, the immune phenotype reversed into an immunosuppressive microenvironment, dominated by the infiltration of Treg cells and macrophages, accompanied by a significant enhancement of APC co-inhibition signatures (Fig. 4G and H).

### SXD exerts antitumor effects via remodeling the immune microenvironment and invigorating CD8^+^ T cell cytotoxicity

**Fig. 5:**
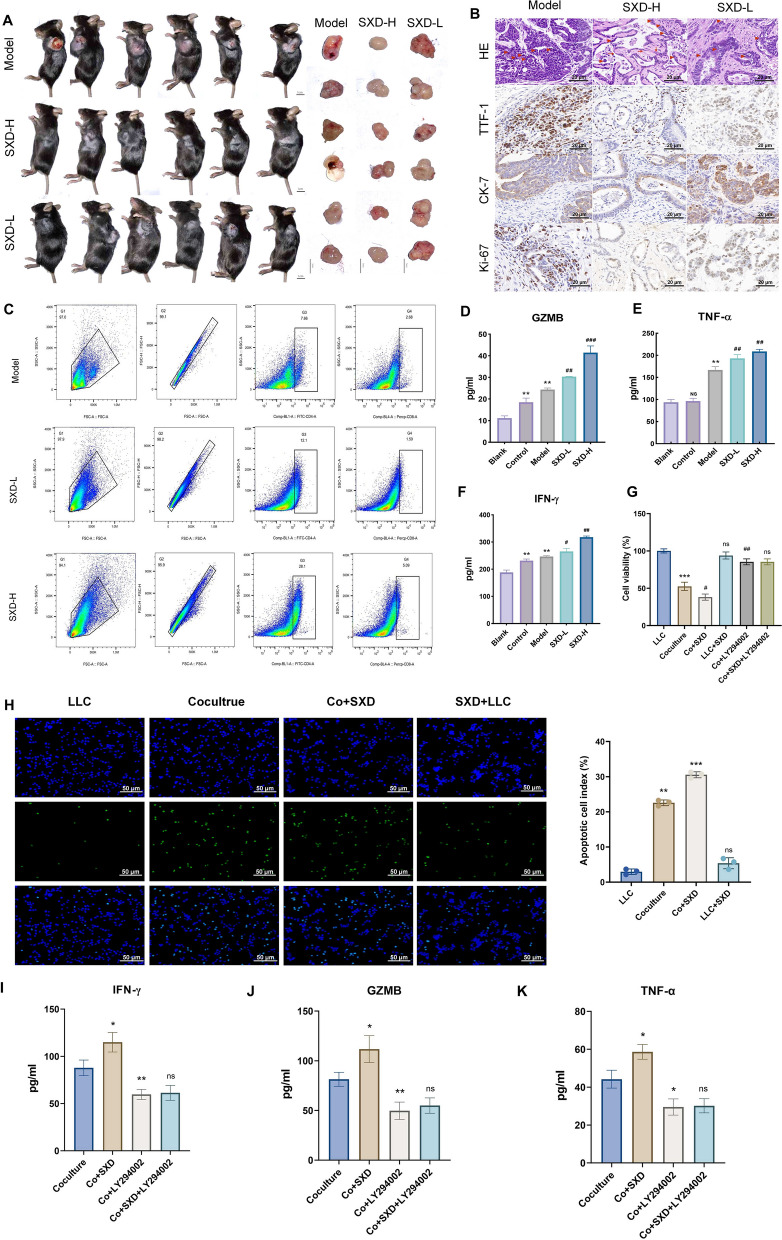
SXD inhibits malignant progression in TK model. **A** bright-field images of the mouse bodies and the subcutaneous tumors at week 10 for the model and Shengxian Decoction low- and high-dose groups. **B** Representative pictures showing H&E and IHC stainingsof TTF-1, CK7, and Ki-67 in tumor sections of the TK mice at week 10 for the model and SXD low- and high-dose groups. Scale bar, 20 μm. **C** Representative flow cytometry plots showing immune cell subsets in tumors. G1 Parent% represents the target cell population of interest. The target population after doublet exclusion. G3 Parent% corresponds to the CD4^+^ T cell subset, and G4 Parent% represents the CD8^+^ T cell subset. **D**–**F** Serum levels of GZMB (**D**), TNF-α (**E**), and IFN-γ (**F**) in Blank, Control, Model, and Shengxian Decoction low-dose/high-dose groups. Data presented as the means ± SD; **P < 0.01 vs. Blank group, ##*P* < 0.01 vs. Model group, calculated by unpaired t-test. **G** CCK-8 detection of survival rate of LLC cells in each group. Data presented as the means ± SD; ***P < 0.001 vs. LLC group, #*P* < 0.05 vs. coculture group. **H** Representative immunofluorescence images (left) and quantitative analysis (right) of LLC cell apoptosis in the coculture system (Scale bars = 50 μm). Blue: DAPI; Green: Apoptotic cells. **I**–**K** Levels of IFN-γ (I), GZMB (**J**), and TNF-α (**K**) in Coculture, Coculture + SXD, Coculture + LY294002, Coculture + SXD + LY294002 groups. Data presented as the means ± SD; **P < 0.01 vs. Coculture group, ns *P* > 0.05 vs. Coculture + LY294002 group, calculated by unpaired t-test

To evaluate the in vivo anti-tumor efficacy of SXD, TK mice were treated with SXD for 10 weeks. Compared to the model group, tumor growth in the SXD-H group was significantly inhibited, with the tumor inhibition rate (TIR) reaching 73.4% at week 10 (*P* < 0.001) (Figs. 5A, S6). The SXD-L group also exhibited an inhibitory effect, with a TIR of 20.7% (*P* < 0.01) (Table S2). In vivo bioluminescence imaging further confirmed that SXD intervention significantly reduced the tumor burden (*P* < 0.01) (Fig. S3 A, B). Furthermore, pathological analysis revealed that the expression of the key tumor marker TTF-1 and the proliferation index Ki-67 were both significantly downregulated in the SXD-H group (*P* < 0.0001) (Figs. 5B, S4). These data indicate that SXD effectively inhibits the malignant progression of lung cancer.

Flow cytometric analysis revealed a robust restoration of immunosurveillance in the SXD group, characterized by significantly elevated proportions of infiltrating CD4^+^ and CD8^+^ T cells (*P* < 0.001, *P* < 0.001), alongside an increased CD4^+^ /CD8^+^ ratio (*P* < 0.001) (Fig. 5C, Table S3). IF analysis visually demonstrated that SXD intervention significantly increased the infiltration density of CD8^+^ T cells in the tumor tissues (*P* < 0.01) (Fig. S5A, B). The immune cell phenotypes validated by flow cytometry and immunofluorescence assays were consistent with the infiltration trends predicted by immune cell infiltration analysis. To further verify the systemic functional status of these effector cells, ELISA results showed that SXD treatment, particularly at the high dose, significantly elevated the serum levels of TNF-α, IFN-γ, and GZMB (*P* < 0.05,* P* < 0.05, and* P* < 0.005) (Fig. 5D–F).

To further determine whether the antitumor effect of SXD is associated with enhanced CD8^+^ T cell function, we performed an in vitro coculture assay using splenic CD8^+^ T cells isolated from mice and LLC tumor cells. Compared with the coculture group, SXD treatment significantly reduced tumor cell viability and increased tumor cell apoptosis (*P* < 0.01) (Fig. 5G, H). Meanwhile, the levels of IFN-γ, TNF-α, and GZMB in the coculture supernatant were significantly elevated (*P* < 0.05,* P* < 0.05, and* P* < 0.005) (Fig. 5I–K), suggesting that SXD treatment enhanced the effector function of CD8^+^ T cells. Notably, in the absence of CD8^+^ T cells, SXD exerted only limited direct effects on LLC cells, with no significant differences in tumor cell viability or apoptosis compared with the LLC group (*P* > 0.05) (Fig. 5G, H). These findings suggest that the antitumor activity of SXD may not primarily depend on direct cytotoxic effects on tumor cells, but rather is associated with enhanced CD8^+^ T cell-mediated antitumor immunity.

### ***FYN*** is identified as a potential target associated with SXD-mediated enhancement of CD8^+^ T cell function in LUAD

**Fig. 6:**
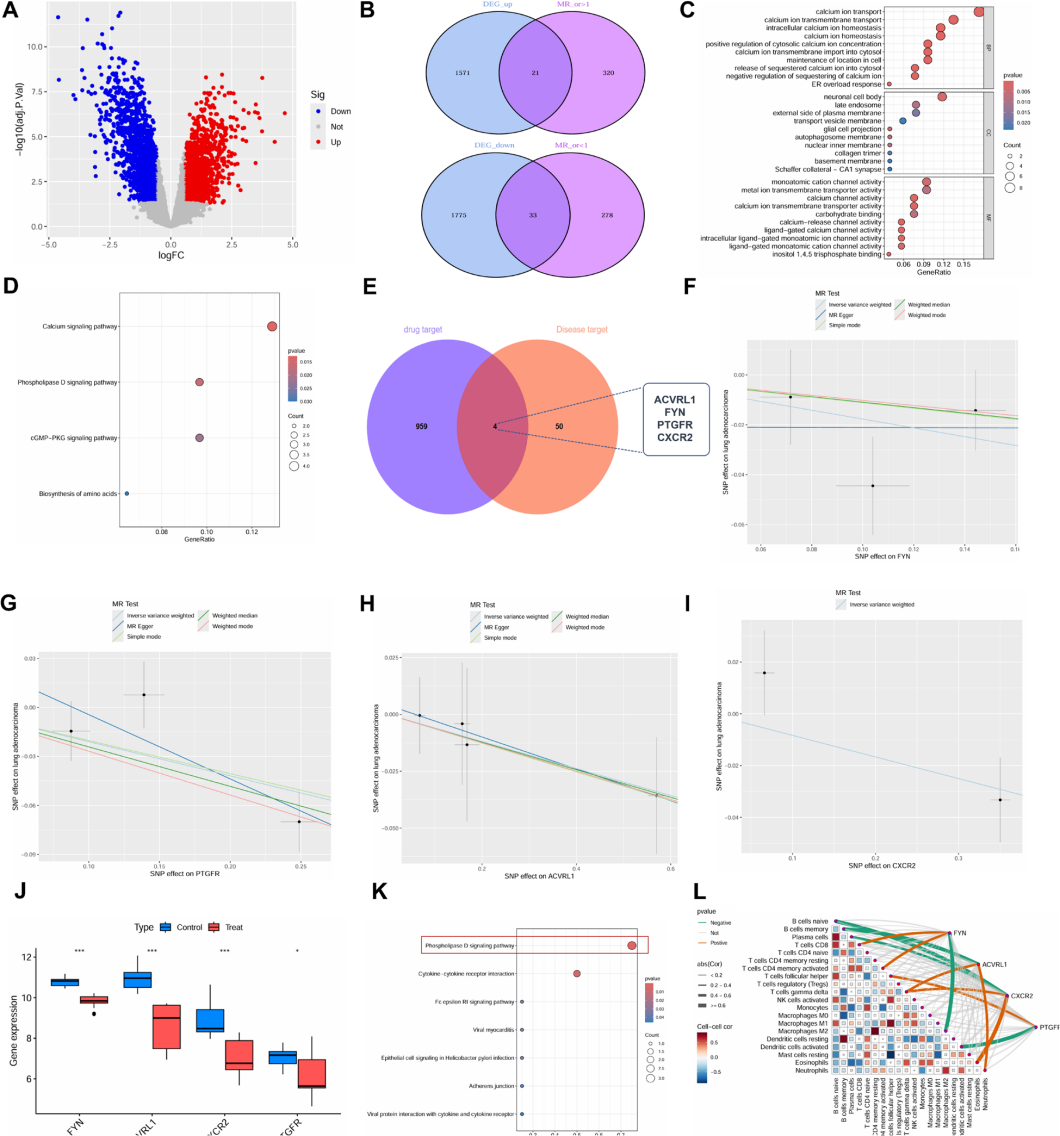
Potential therapeutic targets for LUAD based on network pharmacology and Mendelian randomization analysis using expression quantitative trait loci (eQTL). **A** Volcano plot showing all upregulated and downregulated differentially expressed genes (DEGs) based on GEO datasets. **B** Venn diagram showing key genes: upregulated genes (top) and downregulated genes (bottom). **C** KEGG enrichment analysis showing pathways enriched by the core targets. **D** Gene Ontology enrichment analysis showing core targets in BP, MF, and CC. **E** Venn diagram of core targets. **F**–**I** Scatter plot showing the Mendelian randomization analysis of the causal effects of the core target *FYN* (**F**), *PTGFR* (**G**), *ACVPL1* (**H**), and *CXCR2* (**I**) on disease risk. **J** Expression levels showing the core genes ACVRL1, FYN, PTGFR, and CXCR2 in GEO datasets. **K** KEGG enrichment analysis showing pathways enriched by the core targets. **L** Heatmap illustrating the correlations between 22 immune cell types and the core genes

To investigate the molecular mechanism by which SXD promotes immune function and delays the progression of LUAD, we employed a target identification framework integrating bioinformatics, network pharmacology, and eQTL-based Mendelian randomization (MR) (Fig. S7). The datasets GSE118370 (n = 12) and GSE140797 (n = 14) were selected for analysis. Following preprocessing with Perl and batch effect correction using the sva package in R, the sample distribution shifted from discrete separation toward enhanced overlap (Fig. S8A). Comparison between the experimental and control groups across the two datasets, using a significance threshold of an FDR-adjusted *P* < 0.05, identified 3,400 DEGs, including 1,592 upregulated genes and 1,808 downregulated genes (Fig. 6A). MR analysis further identified 652 genes associated with LUAD (Table S7), and intersection analysis with the DEGs yielded 21 upregulated and 33 downregulated target genes as potential therapeutic candidates (Fig. 6B). Among these, the 21 upregulated genes exhibited positive causal associations with lung adenocarcinoma, whereas the 33 downregulated genes showed inverse causal associations (Fig. S9). Subsequent GO and KEGG enrichment analyses revealed that the GO terms were primarily involved in calcium ion transport, homeostatic regulation, vesicle membrane, nuclear membrane, and collagen-containing basement membrane, while KEGG pathways were significantly enriched in the calcium signaling pathway, phospholipase D (PLD) pathway, cGMP-PKG signaling pathway, and amino acid biosynthesis (Fig. 6C, D).

Meanwhile, we employed UHPLC-MS/MS to characterize the active constituents of SXD, and a total of 148 compounds were identified. Based on ADME criteria screening from the TCMSP database (OB ≥ 30%, DL ≥ 0.18), 71 candidate components were obtained (Fig. S8B, Table S6). Target prediction based on the SwissTargetPrediction, ETCM, and TCMSP databases yielded 964 SXD-related targets. Intersecting the SXD-derived targets with the 54 previously identified LUAD therapeutic targets revealed four core candidate targets: *ACVRL1*, *FYN*, *PTGFR*, and *CXCR2* (Fig. 6E). MR analysis indicated that these four genes were inversely associated with lung adenocarcinoma risk (Fig. 6F–I). In public datasets, the expression of these core genes was significantly downregulated in early-stage lung adenocarcinoma tissues (Fig. 6J), a finding consistent with the MR analysis results. KEGG pathway enrichment analysis showed associations with pathways including the phospholipase D (PLD) signaling pathway and cytokine–receptor interaction (Fig. 6K).

Notably, immune correlation analysis revealed that, among the four core candidate targets, *FYN* exhibited the strongest positive correlation with infiltrating CD8^+^ T cells and activated CD4^+^ memory T cells, while showing a negative correlation with M2 macrophages (Fig. 6L), suggesting its close association with an immune-activated tumor microenvironment. Taken together, these results identified *FYN* as the most likely key target mediating the immunomodulatory effects of SXD in LUAD.

### SXD activates the PI3K/AKT signaling pathway in CD8^+^ T cells in association with ***FYN***

**Fig. 7:**
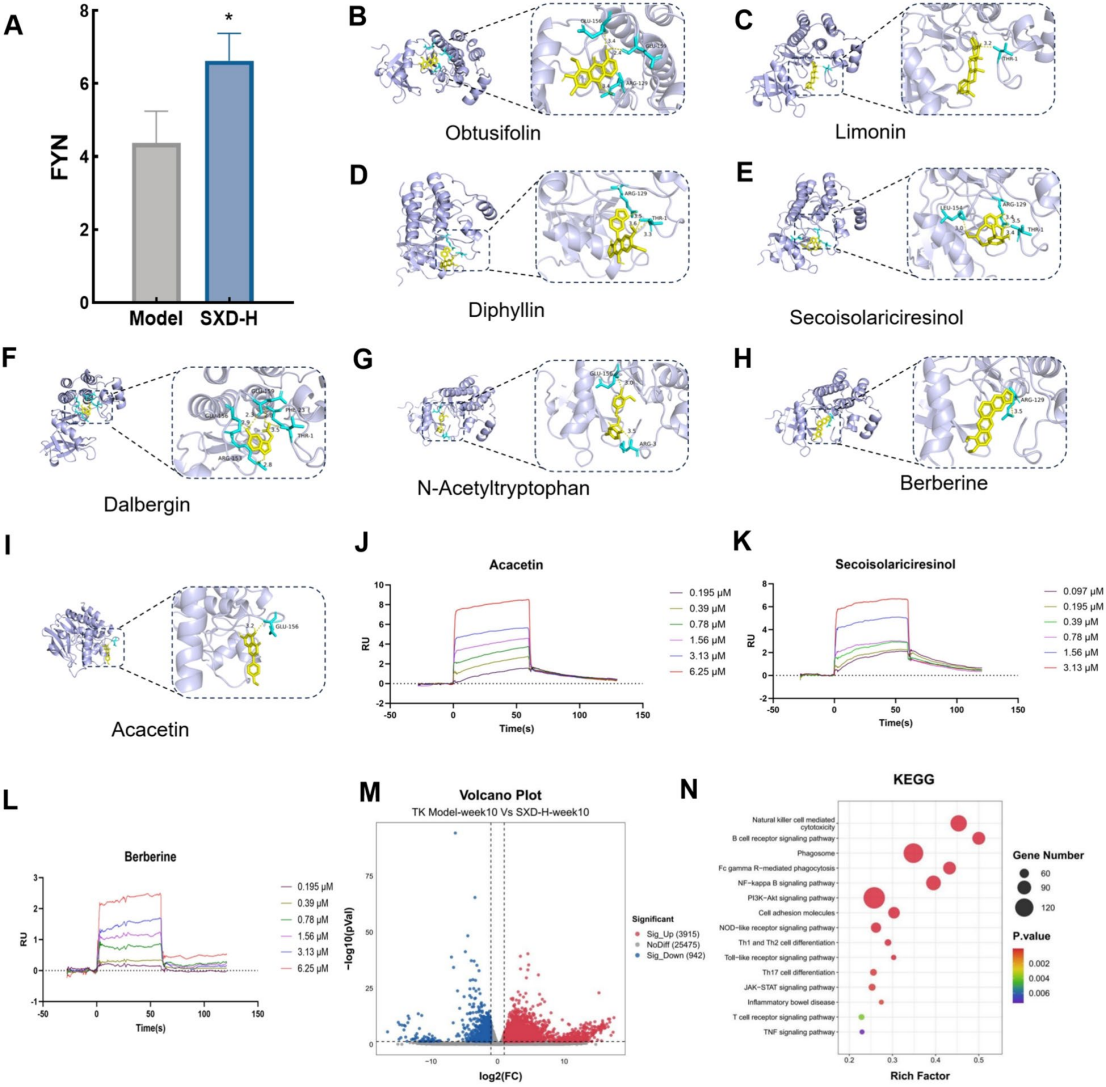
*FYN* as a key target of SXD in enhancing CD8⁺ T cell function and delaying LUAD. **A** Transcriptomic expression of *FYN* in the model group and high-dose SXD group. Data are presented as means ± SD; *P < 0.05, calculated by unpaired t-test. **B**–**I** Molecular docking models of eight representative SXD compounds with the target protein *FYN*.** J-L** Binding affinities of representative SXD active compounds to *FYN* determined by SPR. **M** Volcano plot showing all upregulated and downregulated differentially expressed genes in the high-dose SXD group. **N** KEGG enrichment analysis showing significantly enriched pathways in the high-dose SXD group

After identifying *FYN* as a core candidate target of SXD intervention, we further explored the downstream signaling network potentially mediated by *FYN*. As a key tyrosine kinase in the T cell receptor TCR signaling pathway, *FYN* plays an important role in regulating T cell activation and effector function. Previous studies have shown that *FYN* can modulate T-cell function through classical signaling pathways such as PI3K/AKT [27–29]. Consistent with this, transcriptomic profiling of mouse-derived transplanted tumors revealed that, compared with the model group, *FYN* expression was significantly upregulated in the tumor tissues of the SXD-H group (*P* < 0.05) (Fig. 7A). Concurrently, the PI3K/AKT, as well as NF-κB, JAK-STAT signaling pathway, as well as T cell differentiation-related pathways, including Th1/Th2 and Th17 cell differentiation, were markedly enriched (Fig. 7M, N).

To further evaluate the potential interactions between SXD active constituents and *FYN*, we performed molecular docking analysis. Among the 71 active compounds screened from SXD, 40 were found to form relatively stable binding conformations with the *FYN* protein (Table S4, Fig. 7B–I). Surface plasmon resonance (SPR) assays subsequently validated the direct binding of three representative active constituents—secoisolariciresinol, berberine, and acacetin—to *FYN*, with all exhibiting concentration-dependent binding affinities (Table S5, Fig. 7J–L). Notably, pathway enrichment analysis of the four core targets indicated the involvement of the PLD signaling pathway (Fig. 6K), with PI3K/AKT recognized as a major downstream effector of PLD signaling [30]. Taken together, these findings suggest that SXD may enhance CD8^+^ T cell effector function, at least in part, through modulation of the *FYN*–PI3K/AKT signaling axis, thereby suppressing LUAD progression.

**Fig. 8:**
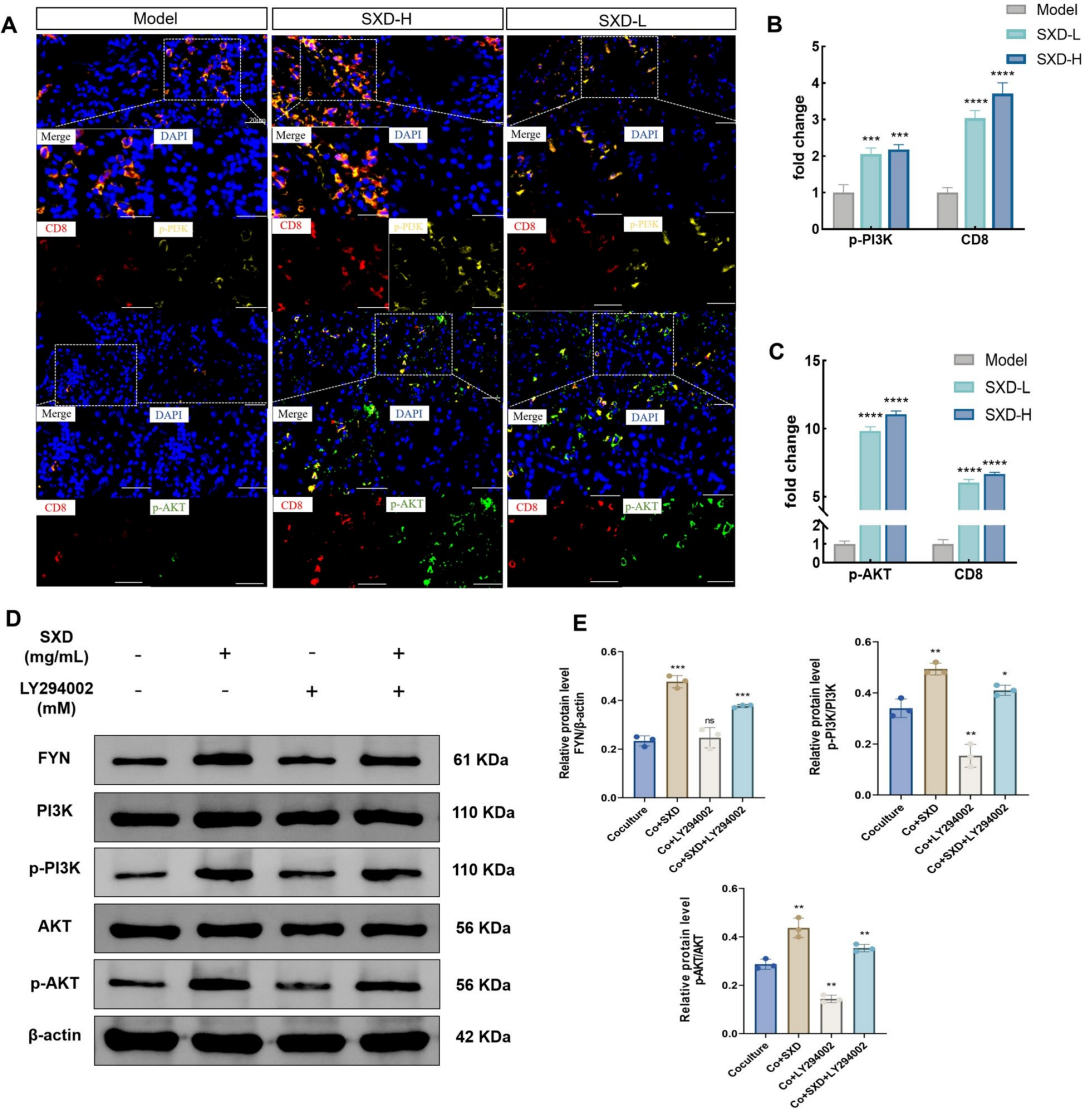
SXD activates the PI3K/AKT signaling pathway in CD8^+^ T cells by targeting *FYN*. **A** Multiplex immunofluorescence staining of PI3K/AKT signaling in CD8 + T cells within tumor tissues from the model group and low- and high-dose SXD groups. **B** Quantification of p-PI3K signaling activity in CD8^+^ T cells across. **C** Quantification of p-AKT signaling activity in CD8^+^ T cells across groups. Data presented as the means ± SD; ***P < 0.001, ****P < 0.0001, calculated by unpaired t-test. **D** Representative Western blot results on the expression of FYN, PI3K, p-PI3K, AKT, and p-AKT. **E** Statistical analysis on FYN/β-actin, p-PI3K/PI3K, and p-AKT/AKT. Data are presented as means ± SD; *P < 0.05, **P < 0.01, ***P < 0.001, calculated by unpaired t-test

To assess the in situ activation status of the PI3K/AKT pathway in CD8^+^ T cells, immunofluorescence staining was performed in tumor tissues. Compared with the model group, the expression levels of phosphorylated PI3K (p-PI3K) and phosphorylated AKT (p-AKT) in CD8^+^ T cells were significantly increased in both the SXD-H and SXD-L groups (*P* < 0.001, *P* < 0.0001) (Fig. 8A–C).

Previous coculture experiments indicated that the antitumor effect of SXD was mainly associated with enhanced effector function of CD8^+^ T cells (Fig. 5G–K). The results of the CD8^+^ T cell and LLC cell co-culture experiment showed that, compared with the control coculture group, SXD treatment increased the expression levels of FYN, p-PI3K, and p-AKT in CD8^+^ T cells, accompanied by significantly elevated secretion of TNF-α, IFN-γ, and GZMB (Fig. 8D–F). To further determine whether this immunoenhancing effect depends on the PI3K/AKT signaling pathway in CD8^+^ T cells, we performed blockade experiments in the coculture system using CD8^+^ T cells pretreated with the PI3K inhibitor LY294002. Following inhibition of the PI3K pathway, the protein expression levels of p-PI3K and p-AKT were markedly reduced were all markedly reduced, indicating effective pathway blockade (Fig. S10). Further comparison showed that, relative to the Co + LY294002 group, FYN expression remained significantly increased in the Co + SXD + LY294002 group, whereas p-PI3K and p-AKT expression showed an increasing trend but did not reach statistical significance. Meanwhile, the promotive effects of SXD on CD8^+^ T cell effector function-related indicators were not fully restored under pathway inhibition (*P* > 0.05) (Fig. 8D–E). Collectively, these findings suggest that SXD may enhance the cytotoxic function of CD8^+^ T cells by upregulating *FYN* expression and activating the PI3K/AKT signaling cascade, thereby delaying LUAD progression.

## Discussion

While lung organoid models offer exceptional controllability and genetic manipulability for disease modeling, their application in capturing the dynamic progression of LUAD remains limited, particularly regarding the in vivo tumorigenic kinetics and the evolution of the TIME following organoid transplantation [31–33]. In this study, by integrating CRISPR/Cas9-mediated gene editing with primary lung organoid transplantation, we established a robust mouse model that recapitulates the dynamic progression of LUAD driven by *Trp53* deficiency and *Kras* activation. Utilizing this immunocompetent model alongside an in vitro co-culture system, we systematically elucidated the antitumor mechanisms of SXD, a traditional Chinese herbal formula. Our findings suggest that *FYN* may represent a candidate target associated with SXD-mediated activation of the PI3K/AKT signaling pathway and enhancement of CD8^+^ T cell function, thereby contributing to remodeling of the TIME and suppression of LUAD progression. This work not only provides a high-fidelity preclinical platform for investigating the early evolution of LUAD but also offers precise scientific evidence for the therapeutic strategy of traditional Chinese medicine in oncology.

Establishing in vivo models that recapitulate tumor evolution is fundamental to mechanistic research [34]. AT2 cells are widely recognized as the predominant cell of origin for *Kras*-mutant LUAD. In contrast to the work of Miura et al. [35], which only observed AAH-like structures in vitro, our TK model—following transplantation into immunocompetent mice—captured for the first time the full spectrum of pathological evolution in vivo. This progression spanned from AAH at week 4, to early IAC at week 6, and finally to advanced IAC at week 10. Furthermore, the expression profiles of molecular markers (e.g., TTF-1, Ki-67) and EMT-associated genes were highly consistent with these pathological stages.

Crucially, the establishment of this model in an immunocompetent host allowed us to systematically characterize the chronological evolution of the TIME. This evolution transitioned from an early Type I interferon response to an intermediate anti-tumor inflammatory state mediated by CD8^+^ T cells and NK cells, and finally to a late-stage immunosuppressive state dominated by Tregs and macrophages. This dynamic immune-editing trajectory provides an ideal window for investigating immune escape mechanisms and evaluating immune intervention strategies. While we acknowledge potential microenvironmental differences between subcutaneous and orthotopic models, substantial evidence indicates that subcutaneous transplantation models exhibit consistency with orthotopic models regarding immune cell infiltration characteristics and drug sensitivity [36–38]. Consequently, our model serves as a rational and robust platform for high-throughput screening and mechanistic immune research.

SXD, a traditional Chinese herbal formula, has recently demonstrated promising therapeutic potential in pulmonary diseases such as chronic obstructive pulmonary disease (COPD) and lung cancer [39–41]. Modern pharmacological studies have revealed that its active constituents, such as astragalus polysaccharides and atractylenolide, can promote CD8^+^ T cell infiltration, inhibit the PD-1/PD-L1 pathway, reverse T cell exhaustion, and enhance the expression of effector molecules like perforin and GZMB [42–44]. However, existing studies have predominantly focused on clinical efficacy, and systematic evidence regarding the immune mechanisms underlying SXD-mediated intervention in LUAD progression remains lacking. In this study, utilizing the TK animal model, we confirmed the significant antitumor efficacy of SXD. SXD intervention not only significantly inhibited tumor growth but also arrested tumor development at an early stage.

Mechanistically, the efficacy of SXD is intrinsically linked to its remodeling of the TIME. Our data demonstrate that SXD significantly reversed the tumor immunosuppressive state, evidenced by enhanced intratumoral CD8^+^ T cell infiltration and the systemic upregulation of immune effector factors, including TNF-α, GZMB, and IFN-γ (*P* < 0.05). To elucidate the underlying molecular mechanisms, we integrated network pharmacology, transcriptomics, and MR analysis, identifying *FYN* as a core target of SXD in LUAD intervention for the first time. FYN serves as a pivotal kinase in TCR signaling. Clinically, *FYN* expression is downregulated in LUAD and positively correlated with CD8^+^ T cell infiltration, with bioinformatic analysis indicating that the PI3K/AKT pathway is a key downstream effector of *FYN* [45–47]. Multiplexed immunofluorescence in situ validation confirmed that SXD specifically activates phosphorylated PI3K (p-PI3K) and phosphorylated AKT (p-AKT) within CD8^+^ T cells. Molecular docking analysis further revealed that multiple active components of SXD (e.g., secoisolariciresinol, berberine, and acacetin) exhibit high binding affinity with FYN protein, and SPR validation provided biophysical evidence for this regulatory target. Notably, tumor burden itself constitutes a non-negligible confounding factor in the evaluation of the TIME. As tumors enlarge, exacerbated local hypoxia and central necrosis can non-specifically drive the recruitment of immunosuppressive myeloid cells (e.g., MDSCs and TAMs) and subsequently accelerate T cell exhaustion—processes that may occur independently of direct pharmacological interventions [48, 49]. To demonstrate that SXD directly promotes CD8^+^ T cell cytotoxicity independently of these macroscopic confounding factors, we performed an in vitro coculture assay using CD8^+^ T cells and LLC cells. These experiments confirmed that the antitumor effect of SXD is primarily mediated through enhanced CD8^+^ T cell–mediated immune surveillance rather than direct tumor cell cytotoxicity. Notably, blockade of the PI3K/AKT signaling pathway in CD8^+^ T cells abolished SXD-induced tumor control, indicating that this signaling cascade is indispensable for SXD-mediated antitumor immunity.

From a translational medicine perspective, these findings suggest that SXD is best positioned as a potential adjuvant immunomodulatory strategy. In particular, since the efficacy of immune checkpoint blockade (ICB) is partially dependent on the presence, activation status, and functional persistence of tumor-reactive CD8^+^ T cells, the capacity of SXD to enhance CD8^+^ T cell infiltration and partially reverse the immunosuppressive TIME may be relevant to PD-1/PD-L1–based combination therapeutic strategies. Of note, the present study did not directly test the combination of SXD with ICB, nor did it evaluate therapeutic responses in clinically defined immunotherapy-resistant settings. Therefore, whether SXD can sensitize LUAD to immune checkpoint inhibitors, reduce immune evasion, or improve clinical outcomes remains to be determined through dedicated preclinical and clinical investigations.

Several limitations of this study should be acknowledged. First, the subcutaneous LUAD model used here is suitable for assessing tumor growth and immune intervention, but it does not fully reproduce the orthotopic lung microenvironment, metastatic behavior, or the full spectrum of host–tumor interactions in vivo. Second, some immune-related findings were derived from transcriptomic analyses, public datasets, and computational inference rather than direct functional profiling of all immune-cell subsets in the tumor microenvironment. Third, although molecular docking and SPR supported representative compound–target interactions, these results should still be interpreted as partial rather than comprehensive validation of the predicted multi-component pharmacological network. In addition, while UHPLC–MS/MS identified candidate constituents present in SXD, we did not directly evaluate their pharmacokinetic exposure or tissue distribution after administration. Future studies incorporating orthotopic models, direct immune phenotyping, pharmacokinetic analyses, and combination experiments with immune checkpoint blockade will be important to further define the mechanistic and translational relevance of SXD in LUAD prevention and treatment.

## Conclusion

In conclusion, this study not only presents an innovative tool that accurately recapitulates the malignant evolution and immune dynamics of LUAD but also unveils, for the first time, a novel immunoregulatory mechanism wherein SXD potentiates CD8^+^ T cell cytotoxicity via the *FYN*–PI3K/AKT axis. These findings provide a robust theoretical foundation for the application of TCM in the intervention of early-stage LUAD.

## Supplementary Information

Supplementary material 1.

Supplementary material 2.

Supplementary material 3.

## Data Availability

The datasets used and analysed during the current study are available from the corresponding author on reasonable request.

## Abbreviations

LUAD: Lung adenocarcinoma
SXD: Shengxian Decoction
PI3K: Phosphatidylinositol 3-kinase
AKT: ‌V-akt murine thymoma viral oncogene homolog‌
AAH: Atypical adenomatous hyperplasia
MIA: Minimally invasive adenocarcinoma
IAC: Invasive Adenocarcinoma
TTF-1: Thyroid transcription factor-1
CK7: Cytokeratin 7
WT: Wild type
IHC: Immunohistochemistry
IF: Immunofluorescence‌
RT-qPCR: Quantitative reverse transcription PCR
IGV: Itegrative genomics viewer
STEM: Short time-series expression miner
PPI: Protein–protein interaction
ELISA: Enzyme-linked immunosorbent assay
IFN-γ: Interferon gamma
GZMB: Granzyme B
EMT: Epithelial-mesenchymal transition
HE: Hematoxylin–eosin
MR: Mendelian randomization
UHPLC-MS/MS: Ultra High-Performance Liquid Chromatography-Tandem Mass Spectrometry
