# Supplementary material for: Shengxian decoction suppresses malignant progression of lung adenocarcinoma by enhancing CD8+ T cell function via the FYN-PI3K/AKT axis
Source: Chin Med. 2026 Jul 6;21:179. doi: 10.1186/s13020-026-01442-9 (PMC13335393; doi:10.1186/s13020-026-01442-9)
Supplement: Supplementary file 1 — Supplementary material 1. [file 13020_2026_1442_MOESM1_ESM.docx]

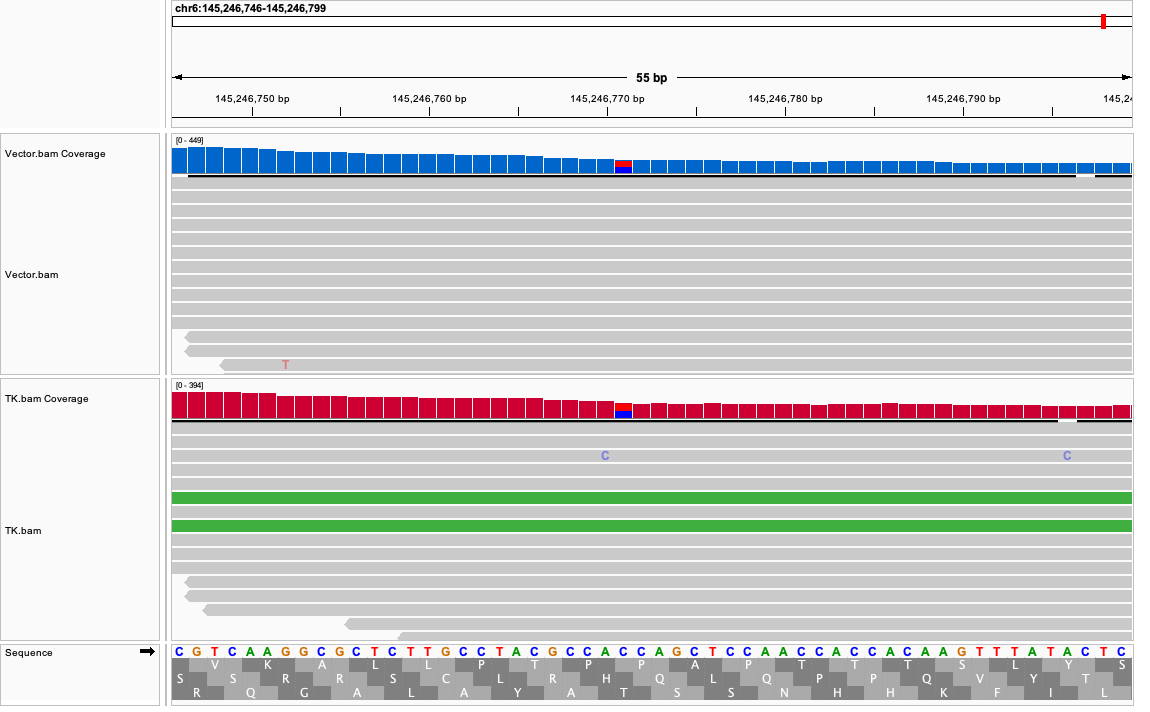


**Fig. S1** Integrative Genomics Viewer exhibiting the mutation site of *Kras* in the TK organoids.


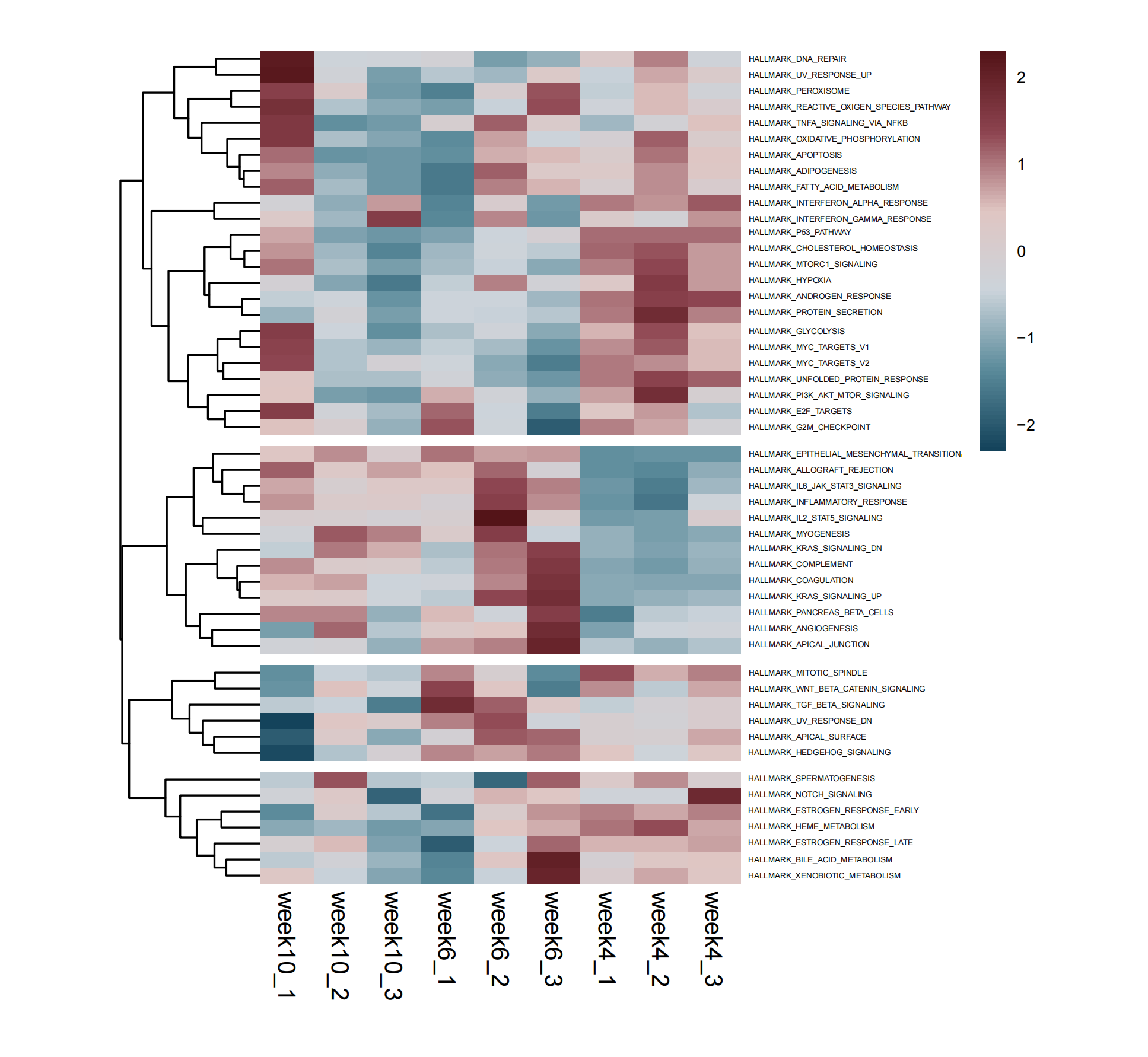


**Fig. S2** Heatmap showing significantly upregulated and downregulated Hallmark gene sets in TK model tumor tissues across week 4, week 6, and week 10.


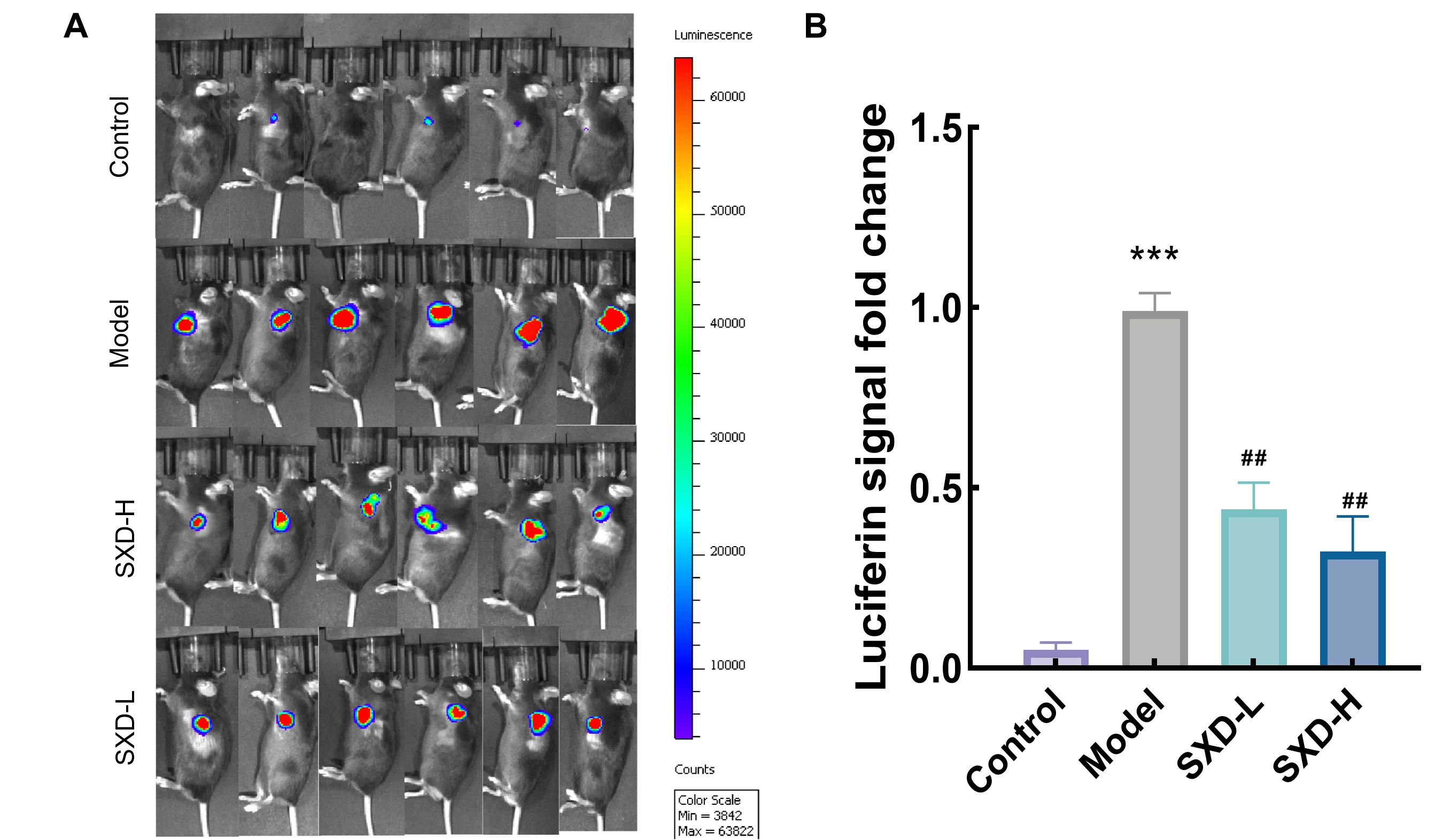


**Fig. S3 A** Representative bioluminescent images of TK mice at week 10 for the model and Shengxian Decoction low- and high-dose groups. **B** Quantification of fluorescence intensity in each group. Data are shown as mean ± SD. **P* < 0.05, ***P* < 0.01 vs. Blank group, #*P* < 0.05, ##*P* < 0.01 vs. Model group, calculated by unpaired t-test.


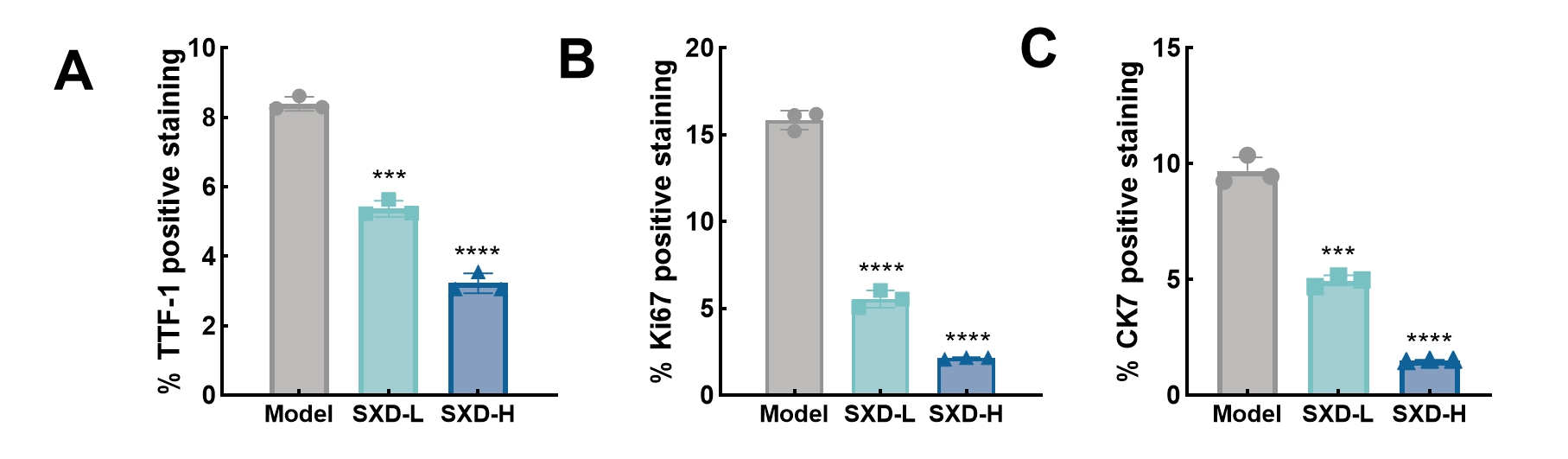


**Fig. S4** **A** Quantification of TTF-1^+^ rates in tumor tissues across groups. Data presented as the means ± the SD; *** *P* < 0.001, **** *P* < 0.0001, calculated by unpaired t-test. **B** Quantification of Ki-67+ rates in tumor tissues across groups. **C** Quantification of CK7^+^ rates in tumor tissues across groups.


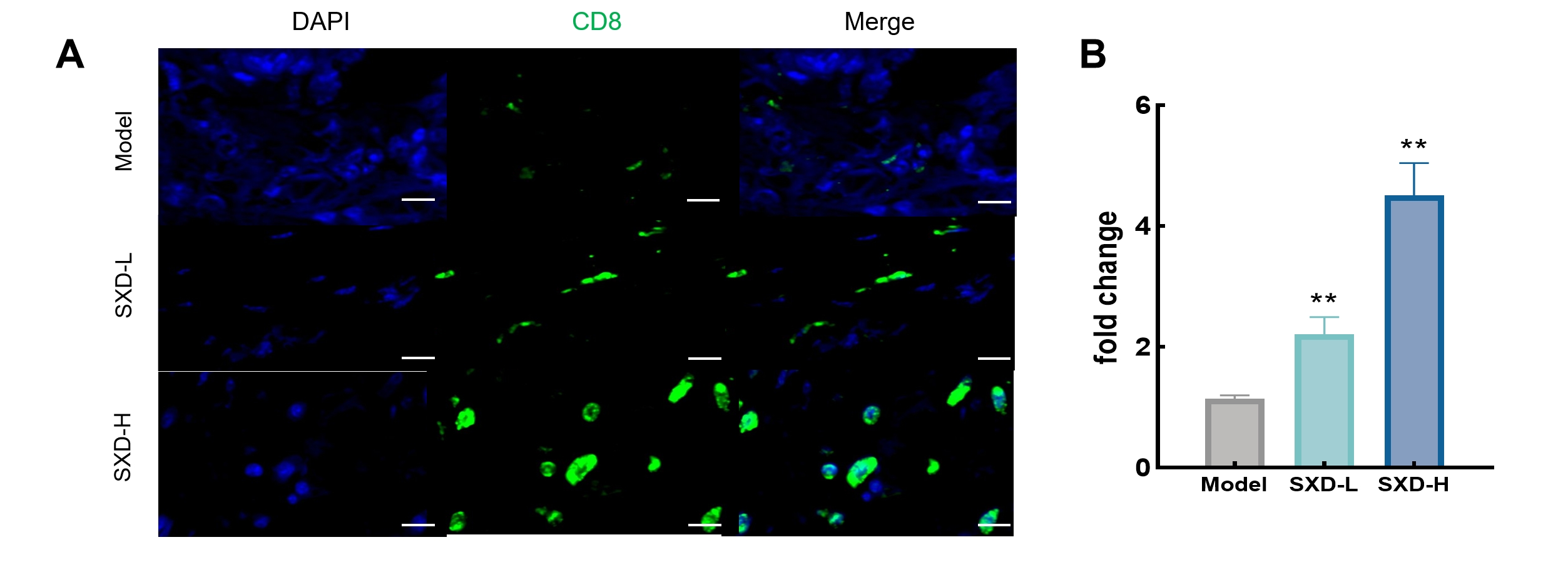


**Fig. S5** **A** mmunofluorescence analysis showing CD8-positive cells in the model group and in the SXD low- and high-dose groups. **B** The fluorescence intensity is presented as fold change relative to the model group. Data are shown as mean ± SD. **, *P* < 0.001, calculated by unpaired t-test.


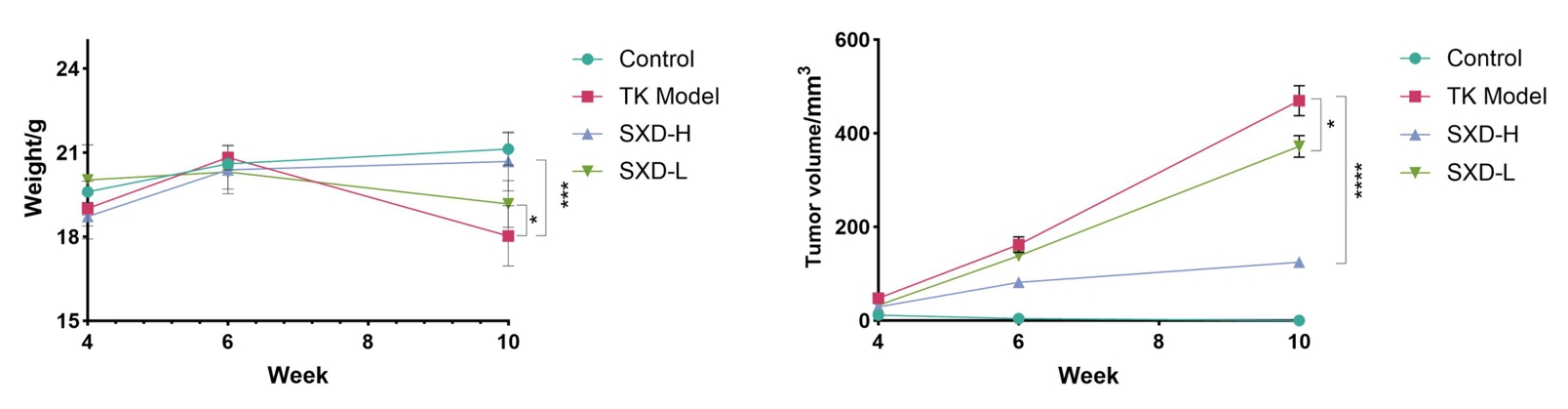


**Fig. S6** **A** Body weight changes of mice in the Control, TK Model, SXD-H, and SXD-L groups during the experimental period. **B** Tumor volume changes in each group from week 4 to week 10. Compared with the TK Model group, SXD treatment, particularly high-dose SXD, markedly suppressed tumor growth while partially alleviating body weight loss. Data are shown as mean ± SD. Statistical significance was determined by one-way ANOVA followed by multiple-comparison test. **P* < 0.05, *****P* < 0.0001.


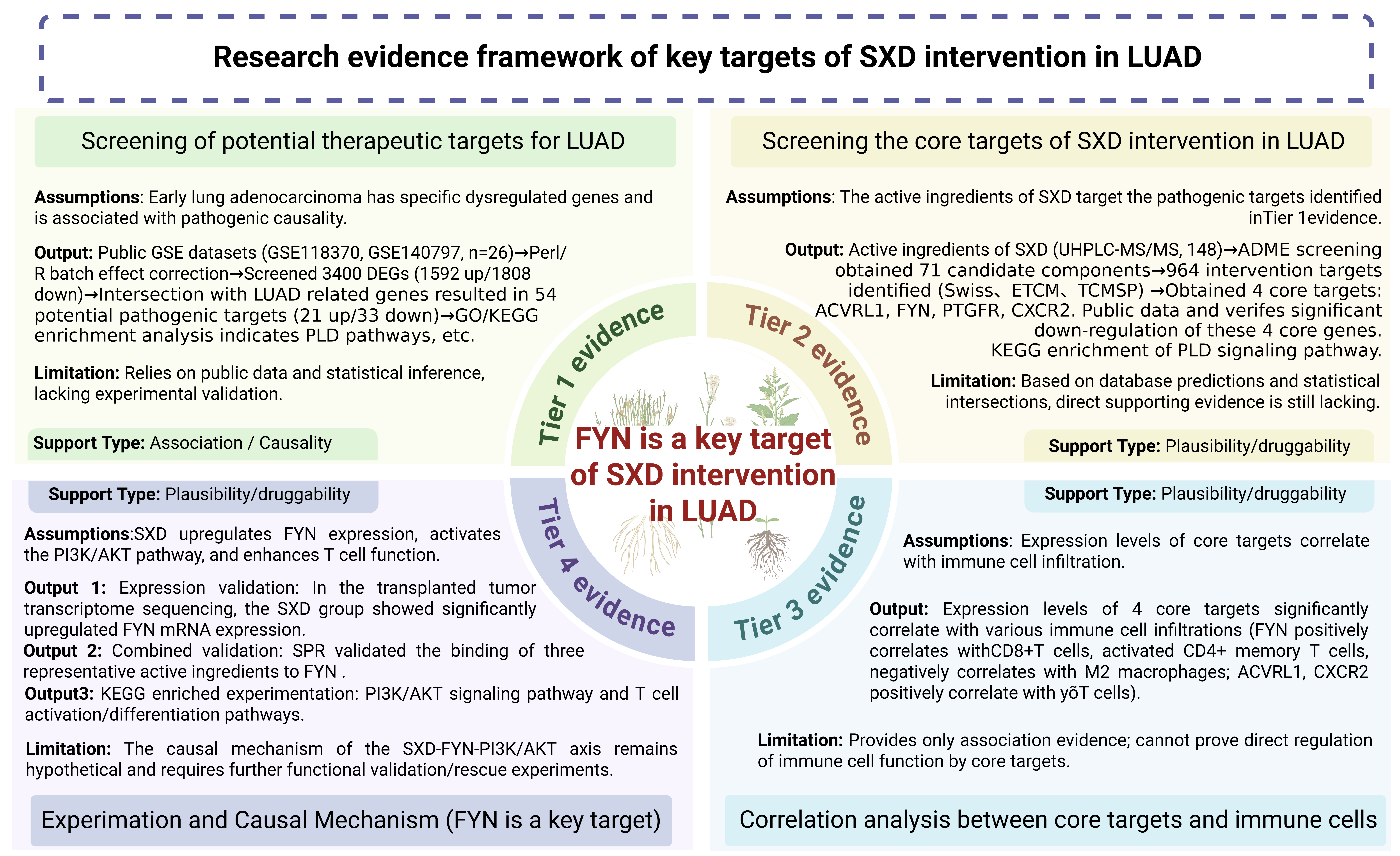


**Fig. S7** Evidence framework for SXD intervention on key targets of LUAD


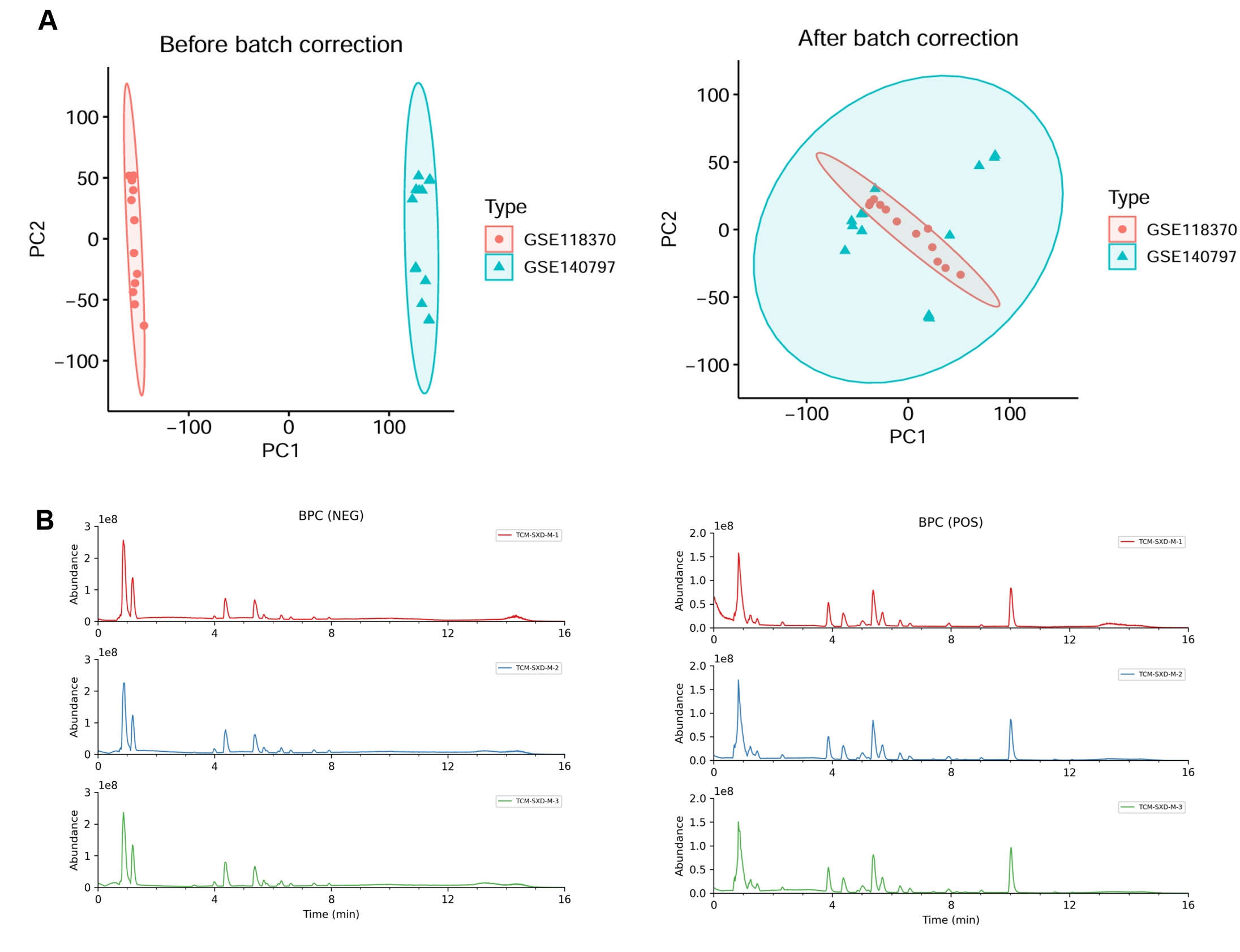


**Fig. S8 A** PCA of gene expression data from GEO before and after batch effect correction. **B** Total ion chromatograms (TICs) of Shengxian Decoction analyzed by UPLC-MS/MS in positive and negative ion modes.


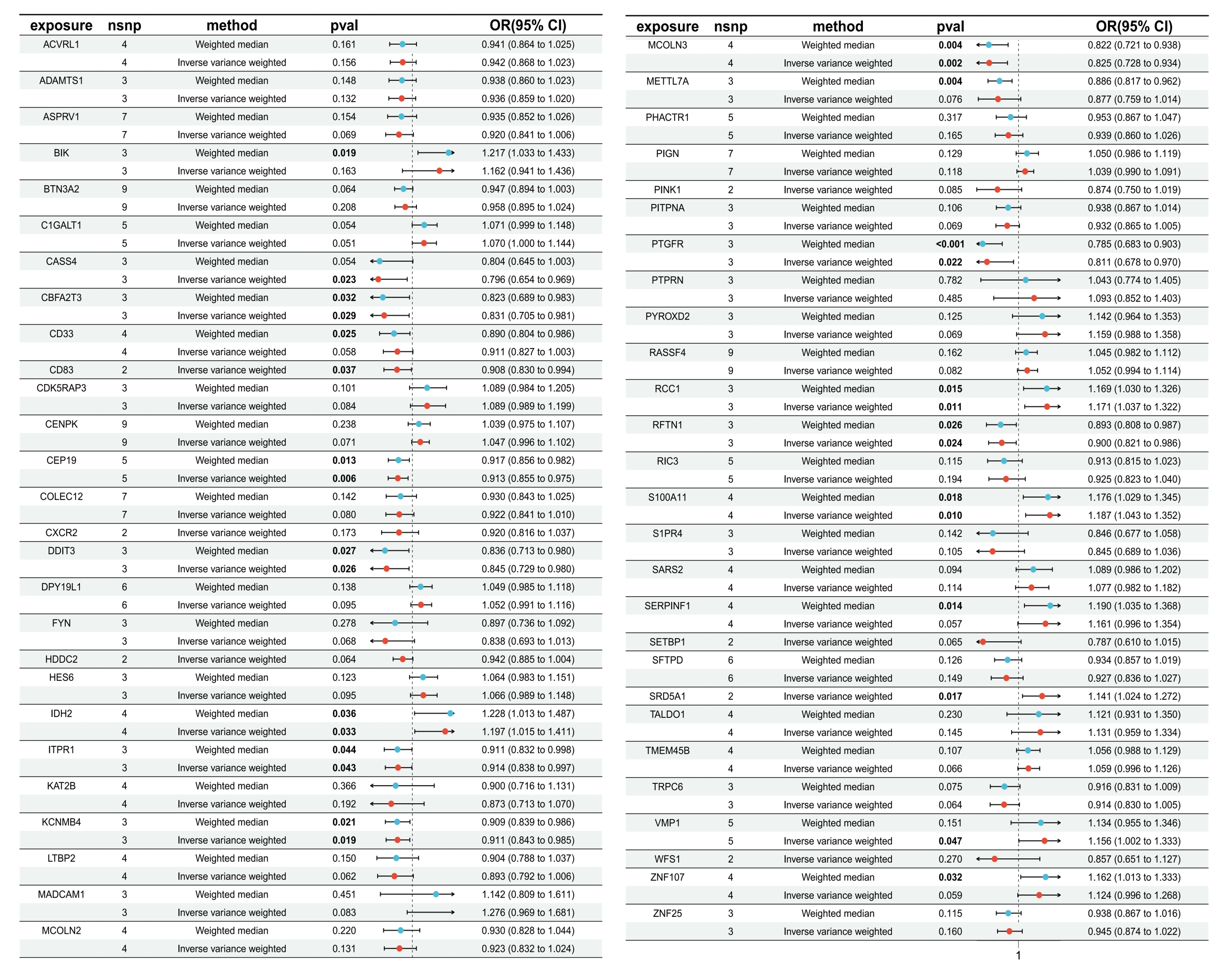


**Fig. S9** Forest plot showing Mendelian randomization analysis for hub genes.（Genes on the left of the OR line are negatively associated with lung adenocarcinoma, while those on the right are positively associated. *p* values in bold indicate significance, *P* < 0.05）


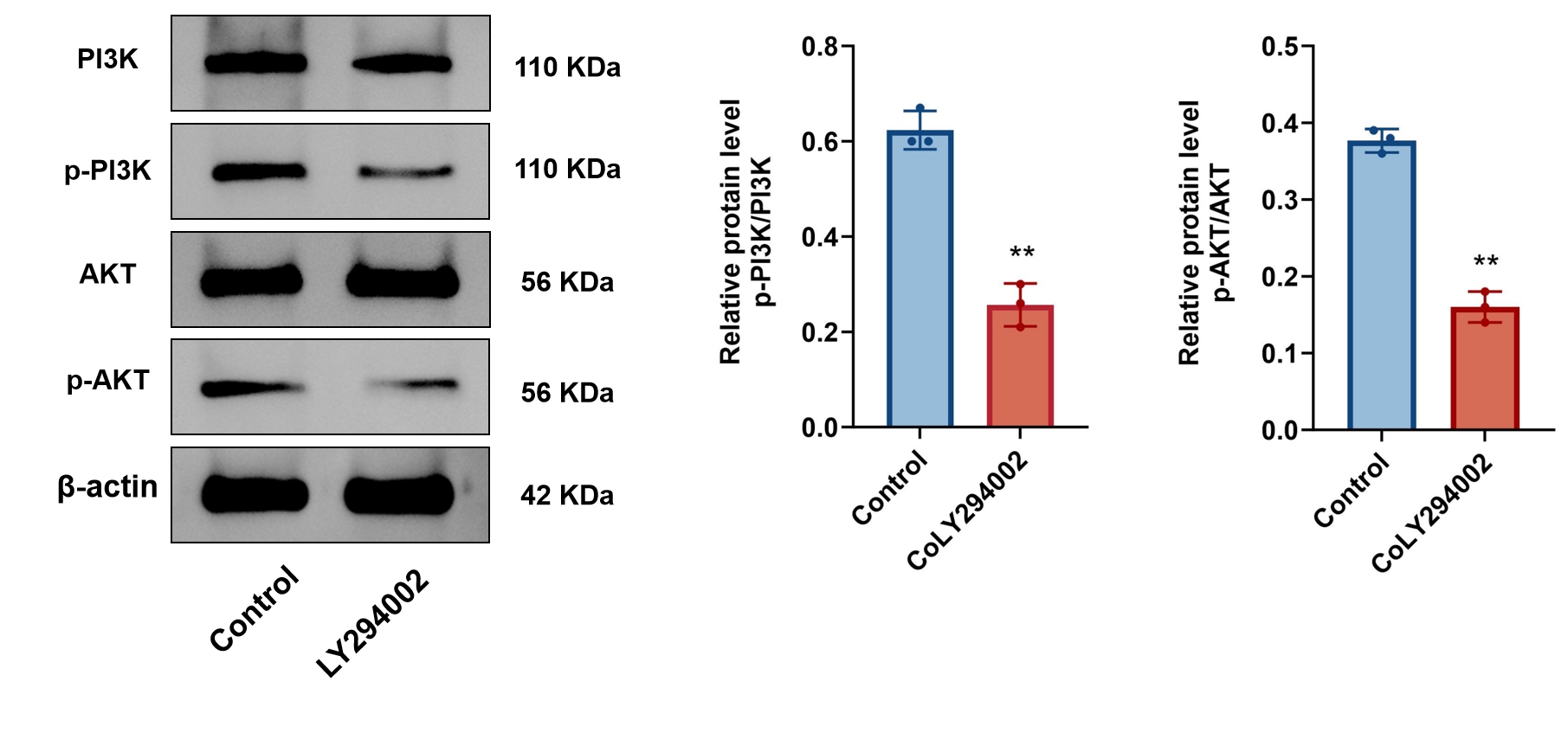


**Fig. S10** Representative Western blot results on the expression of PI3K, p-PI3K, AKT, and p-AKT(left). Statistical analysis on FYN/β-actin, pPI3K/PI3K, and p-AKT/AKT(right). Data are presented as means ± SD; **P* < 0.05, ***P* < 0.01, ****P* < 0.001, calculated by unpaired t-test.

**Table S1.** Tumorigenesis in Control and TK Mice.

| Groups | Week 10 |
| --- | --- |
| Control | 0（0/20） |
| Model | 100%(20/20) |

**Table S2.** Tumor Volume Dynamics and Tumor Inhibition Rate (TIR) in TK Mice (mm^3^, ‾X±SD).

| Groups | Week 4/% | Week 6/% | Week 10/% |
| --- | --- | --- | --- |
| Blank | - | - | - |
| Control | 11.82±0.81 | 4.37±0.89 | - |
| TK Model | 47.94±9.24 | 162.45±16.93 | 469.60±32.12 |
| SXD-H | 29.20±7.90 （39.0%）^**^ | 81.77±5.90^***^ （49.7%） | 124.50±7.99^***^ （73.4%） |
| SXD-L | 32.72±4.03^**^ （31.7%） | 138.30±9.38^*^ （14.9%） | 372.23±23.29^**^ （20.7%） |

**P*  < 0.05, ***P* < 0.01 and ****P*< 0.001 vs. the TK Model group, calculated by unpaired t-test.

**Table S3** Proportion of CD4+T, CD8+T, CD4+/CD8+T cell subsets in TK mouse tissues of each group (‾X±SD,% )

| Groups | CD4+  Parent% | CD8+  Parent% | CD4+/CD8+ |
| --- | --- | --- | --- |
| Model | 7.54±0.35 | 2.17±0.15 | 3.06±0.16 |
| SXD-H | 27.5±0.60^***^ | 5.23±0.25^***^ | 5.27±0.15^***^ |
| SXD-L | 10.78±0.47^***^ | 4.09±0.17^***^ | 3.72 ±0.20^***^ |

**P*  < 0.05, ***P* < 0.01 and ****P*< 0.001 vs. the Model group, calculated by unpaired t-test.

**Table S4.** Binding Affinities Between the 40 Active Compounds of Shengxian Decoction and the Target Protein *FYN*

| Compound Name | Binding Affinity （kcal·mol−1） | Compound Name | Binding Affinity （kcal·mol−1） |
| --- | --- | --- | --- |
| Obtusifolin | -6.6 | Glutamylleucine | -5.4 |
| Limonin | -6.5 | Pratensein | -5.4 |
| Diphyllin | -6.4 | 2-Methoxybenzoicacid | -5.3 |
| Secoisolariciresinol | -6.1 | 3,3-dimethylpentanedioate | -5.3 |
| Dalbergin | -6 | Columbamine | -5.3 |
| N-Acetyltryptophan | -6 | Isofraxidin | -5.3 |
| Acacetin | -5.8 | Serylphenylalanine | -5.3 |
| Berberine | -5.8 | Calycosin | -5.2 |
| Demethyleneberberine | -5.7 | Glycyl-Phenylalanine | -5.2 |
| L-Tryptophan | -5.7 | Methylnissolin-3-O-glucoside | -5.2 |
| Visnagin | -5.7 | Phellodendrine | -5.2 |
| Liquiritigenin | -5.6 | Aspartyl-Leucine_ | -5.1 |
| Phthalicacid | -5.6 | Colutehydroquinone | -5.1 |
| Valylleucine | -5.6 | Cordycepin | -5.1 |
| 3-Coumaricacid_ | -5.5 | Isoleucyl-Valine | -5.1 |
| 5-Hydroxyindoleaceticacid | -5.5 | N-Acetylleucine | -5.1 |
| Hydroxygenkwanin | -5.5 | N-Feruloyltyramine | -5.1 |
| Isomucronulatol | -5.5 | Ononin | -5.1 |
| Methylnissolin | -5.5 | Glycylleucine | -5 |
| 2'-O-Methylisoliquiritigenin | -5.4 | Methylcaffeicacid | -5 |

**Table S5.** Interaction affinity between representative active ingredients of SXD and protein FYN

| Ligand | Analyte | K_D_ (M) | Ka (1/Ms) | Kd (1/s) |
| --- | --- | --- | --- | --- |
| FYN | Acacetin | 8.92e-06 | 5.28e+04 | 4.71e-01 |
| FYN | Berberine | 4.09e-06 | 8.31e+04 | 3.40e-01 |
| FYN | Secoisolariciresinol | 4.84e-07 | 5.17e+04 | 2.50e-02 |

KD: dissociation constant, which reflects the binding affinity of the analyte for the target; a smaller value indicates stronger affinity. Ka: association rate constant, which represents the rate at which two molecules bind; a larger value indicates faster binding. Kd: dissociation rate constant, which represents the rate at which two molecules dissociate; a larger value indicates faster dissociation.
